# Macroautophagy and Mitophagy in Neurodegenerative Disorders: Focus on Therapeutic Interventions

**DOI:** 10.3390/biomedicines9111625

**Published:** 2021-11-05

**Authors:** João Duarte Magalhães, Lígia Fão, Rita Vilaça, Sandra Morais Cardoso, Ana Cristina Rego

**Affiliations:** 1 CNC—Center for Neuroscience and Cell Biology, University of Coimbra, 3004-504 Coimbra, Portugal; joaoduartemagalhaes7@gmail.com (J.D.M.); ligia.fao@outlook.com (L.F.); ritavilacawork@gmail.com (R.V.); 2 Institute for Interdisciplinary Research, University of Coimbra, 3030-789 Coimbra, Portugal; 3 CIBB—Center for Innovative Biomedicine and Biotechnology, University of Coimbra, 3004-504 Coimbra, Portugal; 4 Faculty of Medicine, University of Coimbra, 3000-354 Coimbra, Portugal

**Keywords:** neurodegenerative disorders, autophagy, mitophagy mitochondrial dysfunction, mutant proteins, therapeutic strategies

## Abstract

Macroautophagy, a quality control mechanism, is an evolutionarily conserved pathway of lysosomal degradation of protein aggregates, pathogens, and damaged organelles. As part of its vital homeostatic role, macroautophagy deregulation is associated with various human disorders, including neurodegenerative diseases. There are several lines of evidence that associate protein misfolding and mitochondrial dysfunction in the etiology of Alzheimer’s, Parkinson’s, and Huntington’s diseases. Macroautophagy has been implicated in the degradation of different protein aggregates such as Aβ, tau, alpha-synuclein (α-syn), and mutant huntingtin (mHtt) and in the clearance of dysfunctional mitochondria. Taking these into consideration, targeting autophagy might represent an effective therapeutic strategy to eliminate protein aggregates and to improve mitochondrial function in these disorders. The present review describes our current understanding on the role of macroautophagy in neurodegenerative disorders and focuses on possible strategies for its therapeutic modulation.

## 1. Overview of Autophagy

Autophagy is an important intracellular self-degradative process that maintains intracellular homeostasis through the degradation and recycling of toxic macromolecules and damaged organelles [1]. Much of the current knowledge about autophagy was discovered in the yeast model or in non-polarized cells [2]. This process occurs at basal levels in almost all mammalian cells and can be stimulated in response to starvation, providing the cell with the building blocks for new proteins and lipids. Autophagy plays an important role in the clearance of protein aggregates and pathogens and in the regulation of inflammation and immunity [3,4]. For these reasons, deregulation of autophagy has been implicated in several pathological conditions, including neurodegenerative disorders. Autophagy assumes a critical role in neurons since these cells are very sensitive to the accumulation of misfolded proteins. Neurons rely on anterograde and retrograde transport to cope with the metabolic needs and thus the formation of aggregates not only abrogates the correct function of neurons but also interferes in the communication with the surrounding environment. Hence, the interplay between autophagy and neurodegeneration requires a deeper understanding of the regulatory pathways and the multiple steps that are involved in each process.

**Figure 1:**
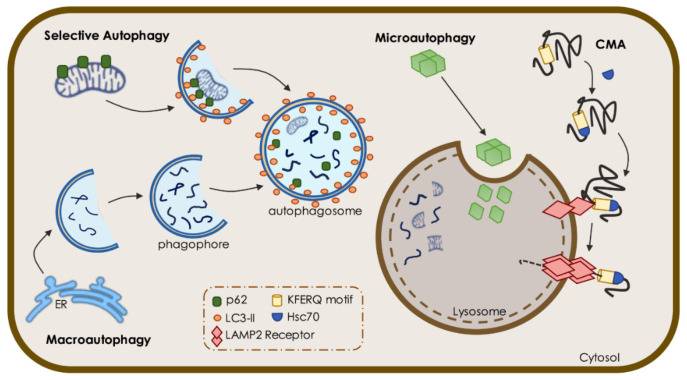
Different types of autophagy. Selective autophagy and macroautophagy initiates with phagophore formation. Through a series of chain reactions, the phagophore sequesters its materials into a double-membrane to form the autophagosome, which, in turn, docks and fuses with the lysosome. In microautophagy, the cargo is directly internalized through invagination of lysosomal and endosomal (not shown) membranes. The chaperone-mediated autophagy (CMA) initiates with recognition of cytosolic proteins carrying KFERQ consensus pentapeptide motifs by the cytosolic HSC70 chaperone and translocation to the lysosomal membrane through binding with lysosomal-associated membrane protein type-2A (LAMP2), resulting in cytosolic protein internalization and degradation.

Autophagy can be divided into three main types, according to the delivery of cargo to the lysosome: macroautophagy, microautophagy, and chaperone-mediated autophagy (CMA). In macroautophagy, the cytoplasmic cargo is engulfed by a growing double-membrane vesicle that, after closure (autophagosome), fuses with the lysosome for degradation (autolysosome) [5]. The process is complex and involves a group of specific autophagy-related proteins acting in a concerted flux, and it can occur randomly (bulk macroautophagy) or selectively through specific adaptors. In microautophagy, the cargo (mostly proteins) is directly internalized through invagination of the lysosome membranes and endosomal vesicles [6]. CMA is a selective process by which proteins with a specific targeting motif (the pentapeptide KFERQ motif) are recognized by cytosolic chaperone heat shock cognate 70 (Hsc70) and its co-chaperones, assisting the translocation of cargo into the lumen of lysosomes through lysosomal-associated membrane protein 2A receptor (LAMP2A) [7]. CMA constitutes an alternative lysosome-mediated degradation pathway that can be upregulated when blockage of macroautophagy occurs [8]. For the scope of this review, macroautophagy and selective autophagy of mitochondria, known as mitophagy, will be further detailed and explored in the context of neuronal function and neurodegeneration (Figure 1).

### 1.1. Macroautophagy

Macroautophagy is a complex and sequential process starting with autophagosome formation and engulfment of cargo, followed by closure and maturation and finally fusion with the lysosome for degradation. Each of these steps involves distinct autophagy-related (ATG) proteins that mechanistically coordinate the autophagic flux along autophagosome biogenesis and fusion with the lysosome [9]. The initiation of autophagy is regulated by the phosphorylation status of unc-51-like autophagy activating kinase 1 (ULK1), which is regulated by the upstream mammalian target of rapamycin complex 1 (mTORC1) [10]. mTORC1 is active in nutrient-rich conditions or when the PI3K/Akt pathway is stimulated by growth factors (e.g., insulin-like growth factor-1, IGF1). Active mTORC1 phosphorylates ULK1 and ATG13, components of the ULK1 initiation complex (also composed by ATG101 and FAK family kinase-interacting protein with 200 kDa, FIP200) [11] suppressing autophagy. In nutrient-depleted conditions, mTORC1 is inactivated, facilitating ULK1 autophosphorylation [12]. Moreover, when the cellular energetic status is low, AMP activates AMPK, which in turn inhibits mTORC1 and phosphorylates ULK1, promoting autophagy [12,13]. Once ULK1 is activated, it phosphorylates ATG13 and FIP200, thus activating the entire ULK1 initiation complex [14]. After ULK1 complex activation, it translocates to omegasomes (specific regions at the endoplasmic reticulum (ER), initiating phagophore membrane assembly for autophagosome biogenesis [15]. At omegasomes, ULK1 promotes the recruitment and activation of class III phosphatidylinositol 3-kinase complex (PI3PK, composed by vacuolar protein sorting 34, beclin-1, phosphoinositide-3-kinase regulatory subunit 4, and ATG14L) through the phosphorylation of Beclin-1 [15,16]. PI3PK is responsible for the generation of phosphatidylinositol-3 phosphate (PI3P) for phagophore expansion [17].

The initial sources of membranes for the phagophore nucleation include COPII vesicles, ATG9 vesicle reservoirs, and the ER-omegasome membrane itself [18]. ATG9 is a transmembrane glycoprotein that cycles between the trans-Golgi network (TGN) and endosomal system through recycling endosomes [19] and is recruited to omegasome upon autophagy induction, thus delivering membranes to the nascent phagophore [20,21]. Moreover, ATG9 shuttles to and from the plasma membrane, the latter through a clathrin-mediated process [22]. Trafficking of ATG9 between membranes is dependent on ULK1-mediated phosphorylation in basal and autophagy-inducing conditions [23]. Other membrane sources, such as the mitochondria, Golgi complex, and plasma membrane, have also been proposed to participate in the vesicle nucleation and expansion [24,25,26]. Specific soluble N-ethylmaleimide-sensitive factor attachment protein receptors (SNAREs) and other tethering factors are involved in the fusion of membranes derived from Golgi, endosomes, or the plasma membrane with the omegasome membrane [15] maintaining vesicle growing.

The generation of PI3P is essential for the nucleation of the phagophore vesicle, recruitment of PI3P-binding proteins that are involved in phagophore expansion, and curvature shaping and recruitment of downstream ATG proteins [27]. The recruitment of PI3P effectors such as WD-repeat domain phosphoinositide-interacting proteins (WIPIs) to the omegasome is essential for autophagosome biogenesis and autophagic flux [27,28]. Alfy, a large scaffolding FYVE domain-containing protein, is a PI3P effector that targets ubiquitinated aggregates to the autophagosome, thus participating in selective autophagy [29]. The next step is the recruitment of microtubule-associated protein 1A/1B-light chain (LC3) to the phagophore, assisted by ubiquitin-like conjugation systems. Initially, the E1 ubiquitin ligase ATG7 and E2 ubiquitin ligase ATG10 are involved in conjugation of ATG12 to ATG5 that further binds to ATG16L1. The complex ATG12–ATG5–ATG161L is essential for the recruitment of the LC3 to the PI3P positive membranes [27]. First, LC3 is proteolytically cleaved at C-terminal by ATG4 protease forming LC3-I, which, in turn, through the action of ATG3 and ATG7 and ATG12–ATG5–ATG161L, generates LC3-II through its binding to the amine headgroup of phosphatidylethanolamine (PE) in the phagophore membrane [30,31]. Such lipidation of LC3 is essential to the expansion and closure of the phagophore and further maturation of the autophagosome [32]. Moreover, lipidated LC3 is involved in specific cargo-recognition via the LIR (LC3 interaction region) domain through selective adaptor proteins [33]. The phagophore expansion and elongation is poorly defined but the interaction of WIPI2 with ATG9-enriched vesicles is essential for the process [21]. Several works showed that additional ATG proteins are involved in the final steps of autophagosome biogenesis, but their exact roles are not clearly defined yet. Studies showed that defects in LC3 ubiquitin-conjugation systems impaired autophagosome closure [34,35], implicating these complexes in the final steps of autophagosome biogenesis. The closure of autophagosome vesicle is mediated by the endosomal sorting complex required for transport (ESCRT) machinery [36]. After the closure, the autophagosome dissociates from ER, and its maturation proceeds through interaction with multiple endocytic vesicles. Autophagosomes can fuse with different endolysosomal compartments, such as late endosomes (LE) and multivesicular bodies (MVB), a feature that varies with cell type and with certain physiological conditions [37]. The fusion of autophagosomes with transient MVBs forms an intermediate structure called the amphisome that will further fuse with lysosome to be degraded [38]. Dephosphorylation of PI3P at the autophagosome membrane by phosphoinositide 3-phosphatases of the myotubularin protein family is required prior to their fusion with lysosomes [39].

**Figure 2:**
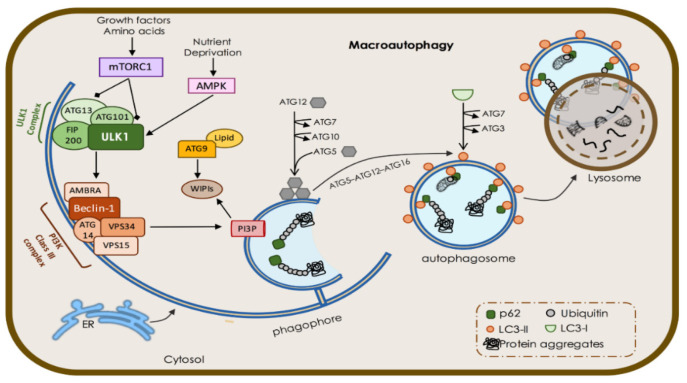
Overview of macroautophagy machinery. Nutrient deprivation and cellular stress induce mTORC1 inhibition and AMPK activation, which, in turn, activate the ULK1 complex. ULK1 complex positive regulation subsequently activates the phosphatidylinositol 3-kinase (PI3K) Class III complex, which leads to phagophore synthesis and initiates autophagy. The sources of membrane for phagophore nucleation and expansion can result from the ER membrane, Golgi apparatus, trans-Golgi network (TGN), plasma membrane, endosomal compartment, or mitochondria. Two ubiquitin-like conjugation systems, the Atg12–Atg5–Atg16L1 complex and LC3-II, participate in the expansion and closure of the phagophore. Once it is completed, the autophagosome can fuse with the lysosome to form the autolysosome, resulting in the degradation of autophagic substrates.

Autophagosomes are randomly distributed throughout the cytoplasm, whereas late endosomes and lysosomes are predominantly found in the perinuclear region but also in neuronal axonal and dendritic compartments [40]. Autophagosomes move along the microtubules towards lysosomes by LC3 and dynein-dependent mechanisms [41]. The fusion of autophagosome with lysosomes is assisted by different protein families. Rab GTPases localize at the vesicle membranes and recruit membrane-tethering proteins that assist SNAREs in the fusion events (reviewed in [42]). Consistently, the depletion of SNARE proteins causes the accumulation of autophagosome in different cells [43,44]. Among the Rab GTPases that regulate autophagosome maturation, Rab7 is recruited to autophagosome membrane and acts as molecular switch, assisting its binding to dynein, thus facilitating the transport of autophagosome and lysosomes towards the perinuclear region [45]. Rab7-knockdown cells display selective impairment of autophagosome fusion with lysosome, but not with LE or MVB [45,46]. Additionally, proteins from the GABARAP subfamily (LC3 homologues) are involved in autophagosome maturation, and their depletion arrests autophagosome–lysosome fusion [47]. Upon fusion, the acidification of autolysosomal lumen by proton pump or v-ATPase activates the lysosomal hydrolytic enzymes [48], resulting in cargo degradation. The resulting metabolites are available for reuse within the cell or act as intracellular signaling molecules (Figure 2).

An essential point of macroautophagy regulation is the post-translational modifications of several ATG and non-ATG proteins (reviewed in [49]). This is essential to limit the extension of autophagy and prevent uncontrolled degradation of cytoplasmic content, which would compromise intracellular homeostasis and may lead to cell death.

### 1.2. Function of Autophagy in Neurons

Neuronal survival relies on both CMA and macroautophagy to balance the levels of misfolded proteins and damaged organelles that otherwise are not able to be diluted through cell division and to sustain cellular processes by recycling metabolites [50]. The ineffective removal of protein aggregates leads to a blockade of axonal transport and major transcriptional alterations; thus, autophagy is essential for maintaining homeostasis throughout the neuronal cell (axon, soma, and dendrites).

In the synaptic regions, the constant protein turnover is indispensable to fulfill the local high energetic demands and to maintain a functional protein synthesis and degradation cycle [51]. This is essential not only to sustain synaptic plasticity [52] but also to support axonal homeostasis [53,54]. Indeed, neuronal stimulation regulates autophagy levels, while autophagy sustains synaptic function at both pre- and post-synaptic domains [55]. Interestingly, apart from the core autophagy machinery, several neural-specific regulatory proteins are required for autophagy at presynaptic regions: Endophillin-A is an endocytic adaptor that generates curved membranes and serves as a platform to recruit autophagic proteins, thus promoting autophagosome biogenesis [56], and synaptojanin 1, a lipid phosphatase that mediates synaptic vesicle trafficking, is also required for autophagosome biogenesis [57]. On the other hand, autophagy can be negatively regulated at presynapses through Bassoon, a scaffold protein that interacts with ATG5, thus making it unavailable for autophagosome biogenesis [58]. At post-synaptic regions, autophagy is essential to sustain synaptic plasticity. In long-term depression (LTD), autophagy is crucial to mediate the trafficking and elimination of α-amino-3-hydroxy-5-methyl-4-isoxazolepropionic acid (AMPA) receptors. Interestingly, stimulation of *N*-methyl-d-aspartate (NMDA) receptors activates the degradation of AMPA receptors by autophagy [59]. Moreover, brain-derived neurotrophic factor (BDNF) suppresses autophagy in hippocampal neurons, facilitating long-term potentiation (LTP) and memory persistence in mice, thus supporting a role for autophagy in synaptic plasticity [60].

Neuronal macroautophagy is an important mechanism that removes faulty intracellular materials, being extremely efficient in neurons, with a rapid clearance of autophagosomes, since a vast number of autophagosomes accumulate in neurons after lysosomal inhibition [61]. In neurons of the CNS and also of the peripheral nervous system (PNS), autophagosome biogenesis starts in neurites and synaptic terminal regions in the distal axon, being then transported back to the cell soma by retrograde movement to fuse with active lysosomes [62]. Autophagosomes undergo maturation as they move from distally (neurite tip) to proximally (cell soma) by engulfing organelles and soluble cargo and by increasing luminal acidification for efficient cargo degradation. In soma, there is a mixed population of autophagosomes with different maturation states that come from distal regions or that are locally generated [63]. Distal-derived autophagosomes are retained within the somatodendritic compartment and are unable to return to the axon, whereas the autophagosomes from soma can move freely between dendrites and the soma [63]. In normal conditions, autophagosomes are hardly detected in neurons as they rapidly fuse with lysosomes, demonstrating that autophagy is highly efficient in neurons’ autophagy induction and autophagosome clearance in [64]. The autophagosomes derived from distal axons contain cytoplasmic content, and at least 10% of the population contains fragments of mitochondria [62], supporting a role for autophagy-dependent degradation of mitochondria.

Macroautophagy is essential for neurite shaping during its growth and also for neural plasticity. Loss of function of ATG16L1, a core autophagic protein, is sufficient to induce defects in mouse brain formation [65]. Additionally, neural-specific deletion of *ATG9* results in defective development of axon tracts and deficient neurite outgrowth in vitro [66]. However, the decline of ATGs and associated proteins with aging [67] leads to a progressive impairment of macroautophagy, which probably contributes to the late onset of several neurodegenerative diseases. Accumulation of autophagosomes results from an imbalance between their formation and degradation [68], which has been described in Alzheimer’s disease (AD), Parkinson’s disease (PD), and Huntington’s disease (HD) [69]. Although genetic mutations of autophagy-related genes are not described as direct causative factors of neurodegenerative diseases, several pieces of evidence support that disturbances in the macroautophagy process and its regulation are involved in neurodegeneration. Indeed, dysfunction of the autophagic process such as impairment of autophagosome–lysosome fusion [64], defective lysosomal acidification [70], or defective autophagosome transport [71,72] result in the accumulation of toxic protein aggregates and malfunctioning organelles, thus contributing to neurodegeneration. Genetic inactivation of *ATG5*, *ATG7,* or *FIP200* in the CNS causes axon swelling and neuron death in mice, resulting in the progressive impairment of motor function [73,74,75]. Moreover, *ATG5*-null mice die within one day of birth due to neuronal loss caused by autophagy impairment [76].

Although several studies evidence the importance of macroautophagy for neuronal survival and function, little is known about its regulation in neurons and glia. Accumulation of aberrant proteins or inclusion bodies are well-described in several neurodegenerative diseases, and alterations in the autophagic activity can affect neuronal homeostasis and survival. Uncovering the roles of autophagy and regulation in neuronal and glia function will be key to pursue novel therapeutic strategies to halt neuronal dysfunction and degeneration.

## 2. Mitophagy

Mitochondria harbor a double-membrane, composed of an inner mitochondrial membrane (IMM) and outer mitochondrial membrane (OMM), separated by the intramembrane space. This organelle configuration is essential for maintaining an electrochemical gradient, allowing mitochondria to generate the necessary amount of ATP to be used by cells [77]. The elimination of defective mitochondria is an essential quality control mechanism to sustain a healthy pool of mitochondria and maintain a viable network in neuronal cells [78]. These cells are more sensitive to mitochondrial defects due to their high metabolic demands; thus, the selective elimination of dysfunctional organelles is vital for reliable neurotransmission. Mitochondria that are irreversibly damaged or become no longer efficient are eliminated by mitophagy, a selective mechanism where specialized receptors recognize flawed mitochondria and mediate cargo targeting to autophagic vesicles [79].

Cells make use of different methods to recycle defective mitochondria, namely ubiquitin-dependent and independent pathways. Majorly, neuronal cells resort to the ubiquitin-dependent mechanism system controlled by PTEN-induced kinase 1 (PINK1)/Parkin to preserve an active and dynamic pool of mitochondria. PINK1 is a mitochondrial protein whose accumulation in the OMM acts as a sensor for maintaining mitochondrial function, since it flags damaged mitochondria for degradation [80]. PINK1 exhibit a mitochondrial-targeting sequence, thus being systematically imported to the IMM by complexing with translocases of the outer membrane 20 and 40 (TOM20 and TOM40) [81], where it is cleaved by the intramembrane serine protease presenilin-associated rhomboid-like (PARL) [82] and therefore maintained at low levels under physiological conditions. Upon a given damage, the molecular interactions between TOM20/40 and PINK1 decrease and PINK1 clusters in the OMM of damaged mitochondria, promoting Parkin phosphorylation and its subsequent recruitment [83]. Parkin, a cytosolic E3 ubiquitin ligase, ubiquitinates a myriad of proteins of the OMM, which are recognized by receptors such as p62, also denominated sequestosome 1 (SQSTM1). p62/SQSTM1 is the ubiquitin-binding receptor responsible for driving ubiquitinated mitochondria to the autophagosome assembly site by interacting with the ATG8 family LC3/GABARAP receptors [84]. These receptors also recognize the LIR motifs of many OMM proteins for specific targeting of damaged mitochondria to the autophagosome assembly site [85].

In neuronal cells, ubiquitin-independent mitophagy orchestrated by two bi-functional proteins, BCL2 and adenovirus E1B 19 kDa-interacting protein 3 (BNIP3L) and BNIP3-like protein X (NIX), is also responsible for the differentiation of retinal ganglion cells (RGCs). BNIP3 and NIX contain a BH3 domain and are mainly located at the OMM. These proteins have a WXXL-like motif with affinity for LC3 and its homologue GABARAP, driving mitochondria to autophagic vesicles. During retinal neurogenesis, a metabolic shift takes place, accompanied by an increase in the expression of both proteins [86]. NIX activity can be mediated by increasing reactive oxygen species (ROS) levels in several cell lines [87], adding a new layer of complexity apart from developmental functions. Indeed, ROS production increases as a consequence of damaged mitochondria [88] and can induce mitophagy [88]. NIX can also regulate the translocation of Parkin to mitochondria upon a depolarizing stimulus, such as CCCP [87]. In addition, BNIP3L and NIX-regulated mitophagy has neuroprotective functions. In a model of brain ischemia, the selective degradation of mitochondria mediated by these proteins was crucial for neuronal survival and function [89]. Additionally, NIX overexpression alone is sufficient to restore mitophagy defects observed in cell lines derived from PD patients [90].

**Figure 3:**
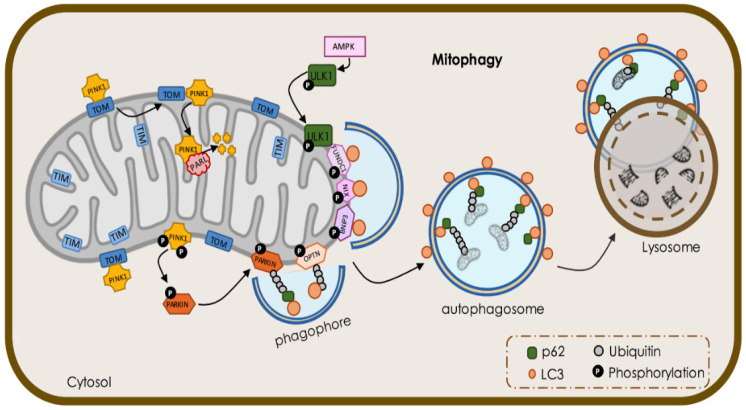
Overview of mitophagy machinery. Dysfunctional mitochondria stabilize PTEN-induced kinase 1 (PINK1) in the outer mitochondrial membrane (OMM) while its proteolytic cleavage through presenilin-associated rhomboid-like (PARL) proteases is blocked. Concurrently, PINK1 recruits Parkin through a series of modifications, including the phosphorylation of both Parkin and ubiquitin. Polyubiquitinated proteins are recognized by several adaptor molecules, including p62 and optineurin (OPTN), anchoring mitochondria to nascent phagophore through microtubule associated light chain 3 (LC3) binding. Receptor-mediated mitophagy relies on various OMM proteins, such as BCL2 Interacting Protein 3 (BNIP3), BNIP3-like protein X (NIX), and FUN14 domain-containing protein 1 (FUNDC1). After elongation is complete, the autophagosome fuses with lysosome for degradation of dysfunctional mitochondria.

Fun14 domain-containing protein 1 (FUNDC1) is an OMM protein that interacts with LC3 and GABARAP and mediates mitophagy in hypoxic conditions. At homeostatic levels, FUNDC1-dependent mitophagy is repressed by Src kinase-mediated phosphorylation in the moiety of the LIR motif at Tyr18. In a hypoxic state, reduced Src activity results in FDUNC1 Tyr18 dephosphorylation, facilitating its interaction with LC3 and subsequent mitophagy [91]. Moreover, hypoxia-driven phosphorylation of ULK1 by AMPK at Ser555 favors ULK1 translocation to the mitochondria, enhancing FUNDC1 activity [92]. Additionally, Optineurin (OPTN) is a cytosolic receptor located at the OMM that promotes mitophagy through its interaction with LC3 [93]. Interestingly, OPTN dysfunction is associated with neurodegenerative diseases, as well as other pathologies [94]. Overall, mitophagy constitutes an essential quality control mechanism for maintaining neuronal metabolism and homeostasis. The transport of mitochondria within neurons relies on anterograde and retrograde movements and is essential to fulfil the energetic and metabolic requirements along the neuronal structure. These movements are also essential for mitochondrial repair and degradation and are assisted by transport adaptor proteins (Figure 3).

## 3. Autophagy and Neurodegenerative Diseases

### 3.1. Alzheimer’s Disease

AD is a neurodegenerative disorder and the most prevalent form of dementia in the elderly population, affecting almost 50 million people worldwide [95]. Early-onset familial AD (EOAD, fAD) accounts for less than 5% of the cases and develops before the age of 65 years. Autosomal dominant mutations in three genes have been associated with EOAD: the amyloid precursor protein (APP) gene and the presenilin-1 (PSEN1) and presinilin-2 (PSEN2) genes, subunits of the γ-secretase complex [95]. However, the most common form is the late-onset sporadic form of AD (LOAD, sAD) with a prevalence higher than 95% and with unknown etiology. The neuropathology of AD is characterized by progressive cognitive impairment and memory decline, which has been correlated with the presence of extracellular deposition of amyloid-β plaques (Aβ, aggregation of β-amyloid peptides) and intraneuronal neurofibrillary tangles (NFTs, aggregation of hyperphosphorylated tau protein) that compromise synaptic integrity and homeostasis, leading to irreversible damage in specific brain regions such as the hippocampus and the cortex [96]. The Aβ peptides are generated by the sequential proteolytic processing of the amyloid precursor protein (APP) by β-site APP-cleaving enzyme 1/β-secretase (BACE1) and γ-secretase through the amyloidogenic pathway. Cleavage of APP by BACE1, the activity of which is elevated in the brain of sporadic AD patients [97], occurs mainly in endosomes, providing an acidic environment for optimal enzyme activity [98]. Intracellular trafficking of APP occurs by secretory and endocytic pathways. APP is secreted to the plasma membrane being then internalized by clathrin-mediated or caveolin-mediated endocytosis [99]. In endosomes, APP can be cleaved by BACE1 giving rise to the N-terminal APP β fragment.

(sAPPβ) and the C-terminal fragment; the latter can undergo further cleavage by γ-secretase, resulting in the formation of Aβ and the APP intracellular domain. In the non-amyloidogenic pathway, APP is cleaved by α-secretase in the middle of Aβ domain, preventing the formation of the Aβ peptides. The cleavage by γ-secretase occurs at different positions of the C-terminal fragment, resulting in the formation of Aβ peptides with different lengths. The most common soluble peptides formed are Aβ1-38 and Aβ1-40, followed by the Aβ1-42, the latter being more hydrophobic and more prone to form trimeric and tetrameric oligomers, thus representing the most toxic isoform [100]. Aβ oligomers tend to form Aβ fibrils with β-pleated sheets that assemble extracellularly into amyloid plaques [101]. Aβ has been also associated with the hyperphosphorylation and aggregation of tau, a microtubule-associated protein that normally assists in the polymerization and assembly of axonal microtubules and vesicle transport. The phosphorylation status of tau is regulated by distinct kinases and phosphatases that are modulated in the presence of Aβ oligomers; for instance, glycogen synthase kinase-3β (GSK3β) and protein phosphatase 2A (PP2A) directly regulate tau phosphorylation status [102]. The hyperphosphorylated state of tau inhibits its association with microtubules, affecting its stabilization and leading to microtubule network disassembly [103,104]. The proteolytic cleavage of hyperphosphorylated tau triggers the formation of tau oligomers [105] that are more toxic than aggregates, leading to progressive neuronal death [106].

#### 3.1.1. Autophagy Impairment in AD

In AD, distinct molecular pathogenic mechanisms resulting from the accumulation of Aβ oligomers and tau hyperphosphorylation have been reported, namely mitochondrial dysfunction [107,108] and disruption of proteostasis systems, such as unfolded protein response and autophagy [109].

Although Aβ peptides exhibit a KFERQ-related motif, which is important for normal processing and degradation of APP, these fragments are not substrates for CMA degradation [110], and macroautophagy assists in their clearance [111]. Indeed, macroautophagy plays an important role in Aβ metabolism because it is involved in the degradation and clearance of APP [112] and its cleavage products, including Aβ [113] and APP-CTFs (amyloid precursor protein cleaved C-terminal fragment) [114].

Autophagy dysfunction has been demonstrated in both AD patients and animal models. In AD patient’s brains, accumulation of immature autophagic vesicles containing filamentous tau was observed in dystrophic neurites [115]. These autophagic vesicles are often found in proximal regions of amyloid plaques in the brains of patients and rodent models [116]. Curiously, accumulation of autophagosomes was also observed in dendrites and soma of APP/PS1 transgenic mouse neurons even before the appearance of Aβ plaques [117]. Additionally, it was observed an accumulation of immature autophagic vesicles prior to neuronal loss in the hippocampus of the *PS1*^M146L^/*APP*^751SL^ mouse model [118]. The abnormal accumulation of autophagosomes in neurons suggests that the defective autophagic–lysosomal pathway contributes to the accumulation of Aβ and tau oligomers in AD [117]. APP and PSEN1 localize in autophagosome; thus, an accumulation of these organelles is associated with an increase in Aβ generation, which in turn disturbs autophagosome membranes [115]. Consequently, the immature autophagosomes described in AD brains and in APP/PS1 transgenic mice may constitute another source for Aβ generation. Brains from *PSEN1*-EOAD patients display lysosomal impairment associated with amyloid pathology and neurodegeneration [119]. It has been suggested that autophagosomes accumulation might result from an impairment of autophagosome–lysosome fusion or defective lysosome digestion [120].

Studies showed alteration of autophagy initiation, specifically a decrease in Beclin 1 expression in the cortex of AD patients and mouse models [121]. The increased activity of caspase 3 in AD patients might explain the excessive cleavage of Beclin 1 [122]. The genetic reduction of Beclin 1 in a transgenic mouse model of AD expressing human APP increased Aβ accumulation, whereas overexpression of Beclin 1 significantly reduced Aβ levels [113]. In addition to Beclin-1, other ATG proteins were downregulated in an age-dependent manner, resulting in an accumulation of Aβ [123]. ATG7, a key protein in the regulation of autophagy, was implicated in AD pathology. The levels of ATG7 were found to be reduced in the cerebral cortex and hippocampus of an AD mouse model, but no changes were detected in temporal cortices on AD patients [124]; *ATG7* knockdown mice exhibited a reduction of Aβ secretion accompanied by an increase of intracellular Aβ [125], which further supports a role for ATG7 in the transport of Aβ peptides to multivesicular bodies to be secreted. The expression of p62 was shown to be decreased in a triple transgenic mouse model, compromising the initial steps of selective autophagy in AD [126]. However, a genome-wide analysis revealed transcriptional up-regulation of positive regulators of autophagy in AD. Consistent with activation of autophagy, an upregulation of ATG protein levels and a higher rate of autophagosome formation and lysosomal biogenesis were observed at the early stages of AD [127]. This controversy suggests differential autophagy regulation in the early and late stages of AD, which should be considered when evaluating the mechanism in different models and when searching for therapeutic strategies.

The presence of immature autophagosomes in dystrophic neurites suggests that retrograde transport of autophagy-related vesicles and their maturation are impaired in AD [128]. In support of this, inhibition of autophagosome delivery to lysosomes leads to their accumulation in neurites with similar morphology, as described in AD [64]. In fact, Aβ oligomers can disrupt the dynein–snapin complex, impairing the axonal vesicle transport, which could contribute to defective delivery of autophagosomes to lysosomes in AD [129]. Degradation of tau, which is primarily located in axons and less found in neurites and soma, occurs through proteasome or by macroautophagy, depending on its oligomerization state. AD-associated tau modifications drive its degradation through macroautophagy when the proteasome-mediated proteolysis becomes impaired by disease progression [130]. Of relevance, the degradation of tau can produce different fragments that bind to Hsc70 being targeted to LAMP-2A by the CMA [131,132]. Still, the accumulation of these fragments in the lysosomal membrane causes the aggregation of tau, which disrupts lysosomal integrity and blocks CMA [132]. The impairment of the autophagic–lysosomal pathway results in the aggregation of tau oligomers, which, in turn, are degraded when macroautophagy is induced with rapamycin [132,133]. Tau is essential for the retrograde movement of autophagosomes towards lysosomes through stabilization of microtubules, a role that is compromised in AD [134]. Hyperphosphorylation of tau and its oligomerization results in the displacement of microtubule machinery and consequently in the impairment of autophagosome movement and maturation, which also contributes to autophagy dysfunction.

Apart from defects in autophagosome transport, an impairment in lysosomal function was also described in AD. PSEN1 is a regulator of lysosomal function acting as a chaperone for the vacuolar-ATPase that acidifies the lysosomal lumen. Mutations in PSEN1 impaired lysosomal acidification and autophagosome–lysosome fusion, resulting in accumulation of Aβ-loaded autophagosomes [135]. Additionally, it was suggested that lysosomal proteolysis is affected in AD; high levels of Aβ, LC3-II, and ubiquitinated proteins were present both in lysosome and autophagosome fractions isolated from the AD transgenic mouse model [136]. Consistent with this, the disruption of lysosomal proteolysis by inhibiting lysosomal cathepsins in healthy neurons results in accumulation of autophagic vesicles with similar morphology to those present in AD patients and transgenic mouse models [64]. Aβ also compromises lysosomal membrane integrity, resulting in the release of cathepsins into cytoplasm [137,138]. The presence of cathepsin D in exosomes isolated from blood of preclinical AD patients [139] supports the hypothesis of an ineffective lysosomal function. Curiously, genetic variations in *Cathepsin D* codifying gene were also described as risk factors for AD [140]. The analysis of the autophagy process in neurons from early to late stages of AD showed an increased expression of autophagy genes at early stages but the lysosomal clearance defect resulted in the accumulation of autolysosomal structures with the progression of disease [141].

Sirtuins (SIRTs) encompass a group of nicotinamide adenine dinucleotide (NAD^+^)-dependent proteins that are regulators of multiple cellular pathways. Among them, Sirt1 has been associated with in autophagy regulation. Lower levels of SIRT1 compromise the deacetylation-mediated activation of downstream autophagy proteins (e.g., ATG5, ATG7, and LC3) [142], which may also contribute to autophagy impairment in AD. In fact, the levels of SIRT1 are decreased in the parietal cortex of AD brain patients and correlate with accumulation of Aβ and tau tangles [143].

Activation of mTOR, the central coordinator of autophagy, was described in AD in response to Aβ accumulation [144]. Aβ increases mTOR activity, which also results in higher tau expression and GSK3β-mediated phosphorylation [145], supporting a role for mTOR in tau-mediated pathogenesis. Additionally, secretion of phospho-tau through exocytotic vesicles by mTOR signaling was observed in the brains of AD patients [146]. Dysregulation of the PI3K/Akt/mTOR pathway has been also implicated in AD pathogenesis [147]. Activation of PI3K by growth factors (e.g., insulin-like growth factor-1, IGF1) promotes the phosphorylation of Akt and indirect activation of mTORC1, promoting autophagy. In AD, Aβ interacts with and inhibits the PI3K/Akt/mTOR pathway [148], leading to activation of GSK3β and increased tau hyperphosphorylation.

Moreover, the transcription factor EB (TFEB), the master regulator of lysosome biogenesis, is a downstream target of mTORC1. A decrease in mTORC1 activity induces dephosphorylation of TFEB and its translocation to the nucleus to activate the expression of autophagy and lysosomal biogenesis-related genes [149]. The levels of TFEB were found to be decreased in the brain samples (hippocampus) of AD patients, particularly decreasing nuclear levels of TFEB in advanced stages of disease [150]. Additionally, TFEB translocation to the nucleus was attenuated in presenilin-deficiency fibroblasts and induced pluripotent stem cell-derived human AD neurons, resulting in reduced CLEAR network activation [151]. However, in a double-transgenic APP/PS1 animal model, increased levels of total and nuclear TFEB were detected in the hippocampus of 8-month-old AD mice with a concomitant increase of its downstream targets, suggesting that TFEB is involved in lysosomal-mediated AD progression [152]. The upregulation of TFEB target genes was also reported in PS1 conditional knockout mice and 5xFAD mice [153]. The activation of the TFEB network might reflect an attempt to compensate for the impaired autophagy in AD animal models. The exact role of TFEB as a master regulator of lysosomal function in AD pathogenesis is far from being fully understood and requires further investigation.

#### 3.1.2. Mitophagy in AD

The accumulation of damaged mitochondria is a hallmark of AD. Mitochondrial dysfunction and the associated bioenergetic deficits and oxidative stress contribute to Aβ aggregation and hyperphosphorylation of tau [154,155], which, in turn, are mediators of mitochondrial defects [156]. Accordingly, Aβ peptides were not toxic to cells without a functional mitochondria (mitochondrial DNA depleted cells) [157]. Exposure of primary cortical neurons to Aβ_1-42_ peptides induces the opening of the mitochondrial membrane transition pore [156]. Moreover, co-exposure of cortical neurons with Aβ and NMDA-mediated activation of NMDAR further induce mitochondrial depolarization and increased mitochondrial calcium retention [158]. Additionally, synaptic terminals exposed to Aβ aggregates displayed mitochondrial dysfunction, higher levels of oxidative stress, and impaired glutamate and glucose transport [159]. Furthermore, samples from brain tissue of tau mutant mice exhibited mitochondrial respiratory defects and higher production of ROS [160]. Accumulation of an N-terminal tau fragment was associated with reduced mitochondrial cytochrome c oxidase activity in human AD brains [161]. Moreover, the depletion in ATP caused by mitochondrial defects activates AMPK and consequently autophagy [162], linking mitochondrial function with autophagy regulation.

The clearance of unhealthy mitochondria by mitophagy is compromised in AD although the mechanism is not fully characterized. Previous studies using post-mortem hippocampal tissues from AD patients and AD iPSC-derived neurons showed lower levels of mitophagy-related proteins, lower levels of PINK1 and BNIP3L/NIX and inactivation of mitophagy initiation proteins, such as phospho-ULK1 [163]. The impairment in mitophagy was also described in APP and tau-overexpression models [164]. The role of defective mitophagy in AD pathogenesis was further supported by an improvement of memory in APP/PS1 mouse models after the correction of neuronal mitophagy [163]. Studies with AD models showed tau insertion into the mitochondrial membrane abrogating Parkin-mediated mitophagy [165]. Moreover, overexpression of Parkin-restored mitophagy in Aβ-treated cells and ameliorated mitochondrial function [166]. Despite an increased translocation of Parkin to the mitochondria, the presence of undigested mitochondria inside lysosomes demonstrates a deficient lysosomal efficiency in AD at early stages [167]. The levels of proteins involved in mitochondrial fission, a step that is essential to isolate damaged mitochondria prior to its engulfment by the autophagosome, are elevated in AD brains [168]. Consistently, altered mitochondrial morphology towards fragmentation was observed in AD, which is aggravated when the levels of Aβ and p-tau increase and these proteins interact with Drp1 fission-related protein [169]. An impairment in anterograde movement contributes to mitochondrial dysfunction displayed in neurons from transgenic AD models [170]. Defective anterograde movement impairs the fusion of old mitochondria with newly formed organelle in soma, thus inhibiting their fusion-mediated repair. Additionally, defective retrograde movement described in AD [129] reduces the removal of defective mitochondria in soma by autophagy. The reduced levels of SIRT1 described in AD also compromise the activation of autophagy proteins, stabilization of PINK1 in mitochondria, and the regulation of mitophagy receptors Nix/BNIP3L and LC3 [171]. Moreover, mitochondrial SIRT3 levels are decreased in AD, resulting in lower FOXO3-mediated activation of p62 [172]. Reduced intracellular levels of NAD^+^ were reported in AD [173], and depletion of NAD^+^ levels could also diminish sirtuins activity. Thus, reduced levels and activity of SIRTs can compromise mitophagy, leading to accumulation of damaged mitochondria in AD.

### 3.2. Parkinson’s Disease

PD is a neurodegenerative disease that results from the degenerative loss of dopaminergic neurons in the *substantia nigra pars compacta* (SNpc), leading to dopamine deficiency [174]. Postural instability, bradykinesia, and gaiting are the typical traits of the disease, often accompanied by hyposmia and marked gastrointestinal complications in the form of gastroparesis and constipation [175]. The histopathological hallmark of PD is the presence of fibrillar aggregates referred to as Lewy bodies (LBs), in which α-synuclein (aSyn) is a major constituent [176]. aSyn is a natively unfolded protein with many attributed functions in neuronal development and synaptic signaling [177]. However, some studies indicate that the lack of wild-type (WT) aSyn is not relevant for basal neuronal function [178], suggesting that PD is not caused by a loss of function of aSyn. Despite intense debate, aSyn oligomers are toxic for neuronal cells [179].

#### 3.2.1. Autophagy in PD

Autophagy was firstly associated with PD when Anglade and colleagues found that the death of dopaminergic neurons in SNpc in post-mortem brains of PD patients was associated with autophagic deregulation [180]. Monomeric aSyn is a short-lived protein and thus its physiological levels are maintained mainly by the ubiquitin–proteasome system (UPS) [181]. However, in the case of aSyn intracellular overload, the clearance of native aSyn by the UPS becomes deficient, thus shifting the elimination of aSyn monomers to the autophagosome–lysosomal pathway (ALP) [182]. Interestingly, a study using mice injected with human aSyn adenovirus in the substantia nigra demonstrated that many autophagic proteins are upregulated in the initial phase of the disease, suggesting an increment in autophagy in this stage [183]. However, post-mortem studies indicate an increase in the levels of LC3 II and a decrease in cathepsin D, pointing to an impaired function in the ALP pathway [121]. Moreover, histological findings demonstrate that LC3 II is often localized within aSyn inclusions [184], which implies not only a defective autophagic process but also that macroautophagy is responsible for the clearance of aSyn aggregates [185].

CMA is strongly impaired in PD, as reduced expression of LAMP2a and Hsc70 is observed in the post-mortem brains of PD patients [121,186]. Interestingly, the remaining LAMP2a-positive vesicles co-localized with aSyn. These observations are consistent with the fact that aSyn bears a KFERQ-like pentapeptide, _95_VKKDQ_99_, with a strong specificity to lysosomal vesicles [187,188]. In aSyn oligomers, this pentapeptide is still available and allows aggregates to bind to the lysosomal receptors, blocking the import of aSyn to the lysosome, which partially explains the defects observed in CMA of neuronal cells in PD [185]. Further studies have demonstrated that post-translational modifications in aSyn also hinder the CMA pathway and contribute to overall cellular toxicity [189]. Indeed, specific inhibition of CMA achieved by the downregulation of LAMP2a appears to be responsible for increased levels of native aSyn in rat cortical neurons [190]. Additionally, the reduction in LAMP2a expression in the nigrostriatal circuit of rats leads to dopaminergic cell death, accompanied by an increase in aSyn protein levels and the increase in LC3 expression [191]. Hence, CMA appears to be an extremely relevant pathway for the degradation of WT aSyn.

Glucocerebrosidase (GBA) is a lysosomal protein responsible for the cleavage of glucocerebroside and glucosylsphingosine in the lumen of these vesicles. GBA deficiency leads to substrate accumulation in the lysosome, with prejudicial effects in the endolysosomal pathway. Interestingly, heterozygous mutations in the GBA locus are considered a major risk factor for development of PD [192]. Homozygous GBA mutant individuals develop Gaucher disease, as a result of which a great part develops parkinsonism [193]. In fact, a link between GBA and aSyn pathologies was found in patients with Lewy Body disorders, a spectrum of diseases with alternative forms of parkinsonism. In these patients, the presence of aSyn inclusions was highly correlated with the presence of mutant GBA in the SNpc of post-mortem brains [194]. Remarkably, in dopaminergic neurons derived from iPSC of heterozygous GBA PD patients, showing decreased activity of GBA, an increase in the number and enlargement of lysosomes were found [195], accompanied by increased levels of aSyn oligomers in PD-derived neurons, when compared to control cells. Moreover, GBA knockdown in rat striatum led to an accumulation of oligomeric aSyn, preceded by a disruption in the autophagic pathway. Beclin-1 might mediate the effects of GBA since its activity is downregulated upon a decrease in GBA activity, with a concomitant reduction in LC3 II [196].

Leucine-rich repeat kinase 2 (LRRK2) is linked to a form of autosomal dominant form of PD, being also a major risk factor for idiopathic PD. Apart from many cellular functions, LRRK2 has a prominent role in autophagy, being involved in several distinct phases of the process [197]. A study using an age-dependent LRRK2 knock-in mouse model showed that midbrain neurons had a higher number of LAMP2a positive vesicles when compared to WT mice [197], suggesting a build-up of lysosomal vesicles. This increase was associated with poorer CMA efficiency since the clearance of lysosomal specific substrates with a KFERQ motif was lower on LRRK2 knock-in midbrain cells. This is consistent with evidence that demonstrates that LRRK2 regulates the activity of a subset of Rab GTPases, which are responsible for membrane mobilization, vesicle assembly, and transportation [198,199]. *G2019S* is the most common mutation in *LRRK2*-associated PD, and mutated LRRK2 is thought to halt the autophagic process. For example, differentiated SH-SY5Y neuronal cells expressing this mutant form of LRRK2 presented much smaller neurites and an aberrant accumulation of LC3 vesicles [200]. Moreover, in mice carrying mutant *G2019S LRRK2*, there was an increment of early and late autophagic vesicles, with detrimental effects on the number of neurons bearing tyrosine hydroxylase (TH) and on cortex neuronal morphology [201]. These observations can be corroborated by the fact that LRRK2 activity is believed to affect macroautophagy via Beclin-1 in a mTOR-independent pathway [202].

Since higher expression of native aSyn is considered a risk factor for the development of idiopathic PD [203], macroautophagy assumes a crucial role in the maintenance of aSyn cellular abundance. Interestingly, findings demonstrate that the expression of mutant forms of aSyn or overexpression of WT aSyn block autophagy [204], conferring a pivotal role to this process in disease progression. Corroborating these observations, Vogiatzi and coworkers found that WT aSyn increased 1.5- to 3.8-fold in PC12 cells after the administration of 3-methyladenine (3-MA), a macroautophagy inhibitor [190]. Additionally, the seeding of aSyn fibrils gives rise to degradation-resistant aSyn inclusions, with a negative outcome in macroautophagy. Cells harboring aSyn aggregates display an autophagosome build-up [205], which can be explained by the fact that aSyn inclusions promote the mislocalization of mATG9, a protein that facilitates membrane trafficking for phagophore formation [204]. Recently, it was observed that A30P aSyn expression in midbrain dopaminergic neuronal primary cultures arrests the autophagic flux, observed by a decrease in autophagosome-associated LC3 protein with a concomitant increase in SQSTM1/p62 levels [206]. Furthermore, in peripheral blood mononuclear cells derived from PD patients, autophagic proteins such as ULK1 and Beclin1 were found to be dysregulated, correlating with aSyn accumulation [207].

The self-renewing nature of autophagy is essential for PD pathophysiology, and the inability to recycle misfolded or aggregated proteins has a profound effect on neuronal cells [61]. Hence, studies suggest that an impaired autophagy could be the etiologic factor in PD pathogenesis. For example, specific ablation of *ATG7*, essential for autophagosome elongation in dopaminergic neurons from aged mice, induces characteristic PD features, such as motor frailty, loss of TH-positive neurons, and aSyn deposition [208]. Moreover, *ATG7* depletion leads to the presence of p62-containing aSyn inclusions in SNpc dopaminergic neurons and a robust increase in polyubiquitinated substrates [209]. Although the defects in autophagy are transversal to PD models, the relation of causality needs to be further investigated.

#### 3.2.2. Mitophagy in PD

Mitochondria play a central role in PD pathogenesis [210]. Mitochondrial failure, including decreased complex I activity, well-described in post-mortem brains of idiopathic PD patients [211,212], together with increased oxidative stress in nigrostriatal neurons [213] and lower mitochondrial capacity of buffering Ca^2+^ [214], are well-known features of PD neurodegenerative process, which highlights the paramount importance of clearing damaged mitochondria in diseased neurons. aSyn oligomers have been described to induce mitochondrial damage. Indeed, both WT and mutant aSyn are imported to the mitochondria in cell lines [215], cultured dopaminergic neurons, and PD patients’ brain [216]. However, A53T aSyn has faster accumulation rates in the mitochondria, impairing the activity of complex I and causing exacerbated ROS production [217]. Furthermore, a study using dopaminergic neuronal cultures showed that aSyn aggregates retaining pSer129 aSyn bind preferentially to mitochondria rather than WT aSyn, leading to impaired oxidative phosphorylation [218]. Indeed, mutant aSyn stimulates mitochondrial fragmentation and promotes the presence of cardiolipin at the surface of mitochondria [219]. Cardiolipin is a danger signal elicited by mitochondria that links to LC3 to initiate mitophagy [220]. Remarkably, aSyn can bind to cardiolipin, thus competing with LC3 and halting mitophagy [219].

The dynamic interplay between Parkin and PINK1 allows for the correct identification of damaged mitochondria. Strikingly, mutations in *Parkin* and *PINK1* genes are responsible for early-onset autosomal recessive forms of PD [221]. Pathological evidence was found in the brains of these PD patients, such as reduced population of neurons in the SNpc and fibrillary gliosis [222]. In PD, Parkin activity upregulates Drp1 function to promote mitochondrial fission and downregulates the function of Mfn1/2 to prevent fusion, resulting in smaller mitochondria that are easier to engulf in phagophores [223]. Interestingly, mutant forms of Parkin fail to localize within the mitochondria after a depolarizing insult, leading to the inhibition of mitophagy [224]. Moreover, proteins localized at the OMM that regulate apoptotic events, such as Bcl-XL and Mcl-1, inhibit Parkin recruitment to mitochondria, blocking mitophagy [225]. Additionally, p62-positive mitochondria prone to selective degradation are reduced in cells expressing mutant Parkin [226], which might happen due to Parkin sequestration into insoluble aggregates [227]. Transgenic mice with absent Parkin display an increase in pSer129 aSyn but not in aSyn total levels [228]. Such ablation is able to produce mild nigrostriatal defects [229], associated with altered mitochondrial morphology [230]. These findings can be explained by the Parkin ability to modulate the phosphorylation state of aSyn at Ser129 [231].

It was demonstrated that PINK1 mutant forms associated with PD fail to accurately recruit Parkin to the mitochondria, resulting in dysfunctional mitophagy [232]. Additionally, PINK1 ablation leads to an increase in the number of fragmented mitochondria [233], a common feature in PD patient’s dopaminergic neurons [234]. Although the numbers of dopaminergic neurons remain unaffected by PINK1 depletion, striatal neurons of *PINK1*^−/−^ mice display a reduced capacity of releasing dopamine [235] and are more susceptible to exogenous stressors as oxidative stress [236]. These observations are accompanied by lower respiration rates in mutant animals and decreased activity of the mitochondrial complex I [237], which can explain the contribution of this mutation to PD. Surprisingly, Parkin or PINK1 depletion alone does not induce overt neuronal degeneration in rodents, which suggests that other Parkin/PINK1-independent mitophagic processes may be taking place [228].

The relevance of mitochondrial dysfunction and hence mitophagy is underlined by the fact that several mitochondrial toxins, including 1-methyl-4-phenyl-1,2,3,6-tetrahydropyridine (MPTP) and rotenone, often mimic the molecular dysfunction in PD and are used as models for idiopathic PD [238]. These toxins target the mitochondria by inhibiting complex I, a feature usually observed in post-mortem brains of PD patients [212]. MPTP induces parkinsonism by promoting dopaminergic neurodegeneration and aSyn accumulation [239]. MPTP is a lipophilic compound that is able to cross the blood–brain barrier (BBB), and in astrocytes, it is converted to its ionic form 1-methyl-4-phenylpyridinium (MPP+) [240]. This active form (MPP+) serves as a high-affinity substrate for dopaminergic transporters to get into neuronal cells [241], inhibiting mitochondrial complex I and consequently reducing respiration rates [239], among other cytotoxic effects. Indeed, MPP+ treatment in primary rat dopaminergic neurons and SH-SY5Y cells promotes Drp1-dependent mitochondrial fragmentation [242]. Additionally, in PC12 dopaminergic-differentiated cells, MPP+-induced mitochondrial damage is accompanied by a dramatic decrease in axonal transport rates, followed by a marked neurodegenerative process [243]. Likewise, rotenone, another strong complex I inhibitor, is able to mimic the phenotypic hallmarks of PD. When injected in mice, rotenone stimulates the selective death of dopaminergic neurons along with the appearance of aSyn aggregates and motor dysfunction [244]. In addition, rotenone administration disrupts the autophagic flux, denounced by the increase in LC3 and p62 and decreased levels of LAMP2a, halting the selective degradation of mitochondria [244]. Of note, rotenone has a severe impact in microtubules network [245], impairing the autophagic flux in rats [244] and altering the movement rates of mitochondria in neurites of differentiated SH-SY5Y cells [246]. Since mitophagy is highly dependent on microtubule-dependent transport [247], mitophagy is expected to be impaired after rotenone exposure.

### 3.3. Huntington’s Disease

Huntington’s Disease (HD) is an autosomal-dominant inherited neurodegenerative disease, characterized by cognitive dysfunction, psychiatric and behavioral disturbances, involuntary motor movement, and later dementia [248,249]. HD selectively causes neurodegeneration of medium spiny neurons (MSNs) in striatum, more specifically, in the caudate and putamen, with a dorsomedial to ventrolateral direction [250]. Later in the disease, the parietal cortex is also largely affected, while neurodegeneration in other brain regions, namely the cerebellum, thalamus, and white matter, has also been described [251]. The genetic cause of HD is a repeat expansion mutation of the trinucleotide cytosine, adenine, and guanine (CAG) in the coding region of the ubiquitously expressed *HTT* gene (with 36 CAG repeats being the pathological threshold), encoding an abnormally long N-terminal polyglutamine (polyQ) tract [252]. The polyQ expansion leads to mutant HTT (mHTT) aggregation, resulting in mitochondrial and synaptic dysfunction, altered mitochondrial Ca^2+^ handling, endoplasmic reticulum (ER) stress, impaired gene transcription and protein translation, inhibited protein clearance pathways, and neuronal loss [253]. Abnormal deposition of misfolded mHTT in nucleus (forming inclusion bodies) and perinuclear/cytoplasmic subcellular regions is another HD feature. These aggregates mostly comprise N-terminal fragments of polyQ-expanded HTT, as shown in neuronal nuclei and dystrophic neurites throughout the cortex and striatum of HD patients [254,255]. These inclusions are enriched in ubiquitin and ubiquitinated-HTT, components of the UPS, and chaperones such as heat-shock proteins [254], reflecting a generalized deficiency in UPS-mediated degradation of mHTT. Indeed, UPS function is diminished in different HD models, which can be accounted for by proteasome sequestration by mHTT aggregates [256].

#### 3.3.1. Autophagy in HD

Two putative KFERQ-like CMA-targeting motifs were identified in the HTT protein: one at amino acids 99–103 (KDRVN), and one at amino acids 248–252 (NEIKV). Upon phosphorylation at Ser16, the very N-terminus (14-LKSFQ-18) is considered a KFERQ-like third motif [257]. While normal HTT is degraded by the UPS, polyQ-HTT is a target for CMA-degradation, since purified intact lysosomes showed mHTT fragments in a LAMP2A-dependent manner [257]. However, CMA-dependent mHTT degradation is less efficient. This may be explained by polyQ expansion-mediated delay of mHTT CMA transport across the lysosomal membrane, leading to accumulation in the cytosol. Furthermore, LAMP2A and Hsc70 can strongly interact with polyQ-HTT, which results in CMA pathway traffic blockage [257]. However, LAMP2 mRNA expression is increased in the caudate nucleus of HD patients, which is consistent with increased levels of LAMP2 protein and concomitant induction of CMA in HD patient cells and mouse models [258], suggesting an attempt to increase the activity of this system. Indeed, full-length polyQ-HTT is primarily targeted to macroautophagy, whereas N-terminal fragments of HTT are selectively targeted to CMA [37]. Interestingly, R6/2 HD mice showed diminished inclusion formation and improved HD phenotypes after N-terminal mHTT fragment target to the CMA pathway. Furthermore, clearance of phosphorylated N-terminal fragments of polyQ-HTT depends on LAMP2A and Hsc70 proteins [259]. Fibroblasts from HD patients, striatal *Hdh* knock-in cells, and pre-symptomatic HTT Q111 knock-in mice showed augmented CMA activity [258]. This increase in CMA activity was not sustained when aged mice became symptomatic. Moreover, a significant decrease in LAMP-2A staining was detected in older HTT Q111 knock-in mice [258]. Since LAMP-2A localization and CMA activity is regulated by lipid composition at the lysosomal membrane, and altered lipid metabolism occurs in HD [260], modifications in lipid homeostasis might contribute to HD dysfunction in CMA. Thus, to compensate for a malfunctioning macroautophagy pathway, HD neurons might upregulate CMA. However, CMA-competent lysosomes are less efficient at degrading substrates over time, while polyQ-HTT interferes with LAMP-2A and Hsc70 cargo uptake and lipids accumulation.

As an attempt to maintain the normal proteostasis, since UPS-mediated protein degradation and CMA fails, macroautophagy may be upregulated in HD [261]. PolyQ-HTT is targeted for autophagic clearance, suggesting selectivity of macroautophagy towards pathogenic HTT [262]. However, UPS and autophagy degradation pathways are significantly compromised in HD, which results in polyQ-HTT aggregates accumulation, ultimately leading to neuronal dysfunction and death [263]. Furthermore, progressive failure of degradation pathways and proteostasis lead to accumulation of damaged organelles such as mitochondria and ER. Interestingly, in addition to polyQ-HTT aggregates being a substrate for autophagy-mediated degradation, HTT protein is further involved in autophagy pathway regulation [264], as described below in this section. Moreover, HTT has a role in axonal transport of autophagosomes, as suggested by live-cell imaging studies in striatal neurons. After both the loss of HTT and the expression of mHTT, decreased autophagosome transport and subsequent inhibition of substrate degradation were observed [265].

Brain biopsies from HD patients showed significant abnormalities in compartments of the vesicular–endocytic pathway, with abnormal proliferation of multivesicular bodies, endosomes and lysosomes, accompanied by disruption of the Golgi apparatus and disorganization of the ER [266]. Concordantly, striatal neurons in brains from HD patients stained for HTT showed significant increases in endosome–lysosome-like organelles and HTT-positive tubulovesicular structures [255].

Normal HTT acts as a scaffold protein to selective autophagy, since HTT can physically interact with p62 in order to facilitate its association with LC3-II and ubiquitinated substrates. HTT can also bind to ULK1, releasing ULK1 from negative regulation by mTOR [267]. Indeed, ULK1 was shown to have reduced activity in HD cellular and animal models (Q175) [268,269]. Moreover, HTT can contribute to the microtubular transport of autophagosomes that is essential for autophagosome–lysosome fusion [265]. Indeed, different cell types, such as primary neurons, striatal cell lines, fibroblasts, and hepatocytes from two HD mouse models and lymphoblasts derived from HD patients showed an increased number of autophagic vacuoles without an augmented autophagy-mediated degradation [260]. Additionally, two HD mice models, R6/2 (which expresses exon 1 of the human *HTT* gene with ~150 CAG repeats) and YAC128 (transgenic yeast artificial chromosome (YAC) mice expressing full-length human *HTT* gene with 128 CAG repeats) mice showed specifically increased levels of the autophagy markers p62 and LC3-II in the striatum at a later age (18 months) [270], suggesting deficit(s) in autophagy-mediated protein degradation. Conversely, polyQ-HTT aggregates can sequester mTOR, leading to autophagy induction [271]. One possible explanation for this contradiction is a substantial alteration in macroautophagy cargo recognition in HD cells, which results in ‘empty’ autophagosomes formation [260]. Autophagosomes have a reduced amount of cytosolic cargo inside, possibly due to abnormal p62–polyQ-HTT interaction, leading to reduced protein degradation rate in HD samples [260], which can result in serious deleterious effects on cell homeostasis and consequent neuronal loss. Compensatory activation of macroautophagy in response to polyQ-HTT aggregates may be an important approach to HD neuron survival. Definitely, organelles are turned over by macroautophagy, requiring the recognition of targeted membranes by the autophagy machinery. Because polyQ-HTT can interact with different organelle membranes [264,271], it is possible that polyQ-HTT interferes with organelle recognition using autophagic vacuoles. Concordantly with this hypothesis, HD cells show accumulation of defective mitochondria, which suggests that compromised mitochondria are not efficiently recognized or marked up to autophagy. Furthermore, polyubiquitination is another way for protein aggregates to be degraded by macroautophagy, and polyQ-HTT can bind to polyubiquitinated protein aggregates, which can restrict their recognition by the autophagy system [262].

Several studies revealed that HTT interacts with autophagy-associated proteins indirectly influencing the autophagy pathway. Clearance of polyubiquitinated inclusions are mediated by the selective autophagy receptor, p62, which can exist in polyubiquitinated mHTT aggregates [272]. Actually, a shell of p62 and LC3 proteins can surround mHTT aggregates to promote recruitment to autophagosomes, whereas p62 knock-down leads to augmented cell death in the presence of mHTT [273]. p62 expression levels are diminished in the HD R6/1 mouse model brain, as a result of decreased protein synthesis, at the early stage of the pathology. However, at late stages, p62 accumulates in the striatum and hippocampus [274]. ST*Hdh*^Q111/Q111^ striatal cells, derived from HD knock-in mice, also showed increased p62 levels, in response to proteotoxic stress [275]. Thus, p62 can bind mHTT aggregates and progressively accumulate in HD mice and patient’s cell nuclei, contributing to the onset of HD symptoms. Previously, Kurosawa and colleagues revealed that p62 depletion in three HD mouse models (R6/2, HD190QG, and HD120QG mice) improved HD phenotypes and life expectancy, showing reduced nuclear inclusions and increased cytoplasmic inclusions of mHTT [276].

The selective autophagy receptor NBR1, which sequestrates protein aggregates, shares a similar function and structure to p62 and has been shown to interact with p62 [277]. As explained before, mHTT forms nuclear inclusions with p62, leading to neurotoxicity. In contrast to what happens with p62, NBR1 does not accumulate within neuronal nuclei or at a late stage of HD in mice or patients [274], suggesting that NBR1 may take over p62 nuclear accumulation to maintain some basic rate of selective macroautophagy. The adaptor protein autophagy-linked FYVE protein (ALFY) acts as a scaffold for aggregates, p62, and the autophagosome and has a central role in the autophagic degradation of mHTT aggregates [278]. Concordantly, ALFY mRNA expression is decreased in the caudate nucleus of HD patients [279]. The autophagy adaptor OPTN can also act as an autophagy receptor, which recognises and promotes autophagic clearance of protein aggregates [280]. OPTN enables intranuclear mHTT inclusion clearance via selective autophagy [281]. Intriguingly, OPTN shows a cell-specific expression pattern in the striatum, which is related to the neuronal loss pattern in this HD brain region [282]. Moreover, OPTN overexpression reduced mHTT aggregation via increased selective autophagy [283]. Toll-interacting protein (Tollip) is another selective autophagy cargo receptor protein with protein aggregate clearance function [284]. A study using the stable Neuro2a cell line expressing mHTT (HD60Q and HD150Q) showed that Tollip associates with mHTT-derived N-terminal peptides, stimulating their aggregation and enhancing their clearance via autophagy, protecting the cells from proteotoxicity [285]. Additionally, the striatum and cortex from the HD R6/2 mice showed Tollip protein in nuclear inclusions [286]. As estimated, Tollip depletion led to cell death, while Tollip overexpression enhanced aggregate clearance [284]. Thus, by acting as a mediator of selective autophagy associated with HD, Tollip might be a good candidate for a HD therapeutic strategy.

Beclin 1 is a major autophagy regulator, the levels of which are reduced with aging. Beclin 1 can be recruited to mHTT aggregates, reducing its activity, further contributing to autophagy dysfunction in HD [287]. A study from Ashkenazi and colleagues, using the HD-N171-82Q mice (expressing the first 171 amino acids of human HTT) and primary fibroblasts from HD patients, showed that mHTT outcompetes ataxin 3 in binding Beclin 1, avoiding its activation [288]. Rhes is a striatal-specific protein and a Beclin 1 activator that recruits Beclin 1 away from Bcl-2, preventing its inhibitory action. However, mHTT can inhibit Rhes interaction with Beclin 1, reducing its role in promoting autophagy [289]. Conversely, Beclin 1 overexpression increased mHTT aggregates clearance and reduced neuronal damage [287]. Expression of Beclin-1 in the human brain gradually decreases with age [287], as well as age-related decreased trafficking of the lysosomal protein LAMP2 [8]. Interestingly, HD patients fibroblasts follow the same pattern [288]. However, Beclin-1 seems to accumulate into polyQ-HTT inclusions in R6/2 HD transgenic mice and HD patients brains [287], demonstrating a region specific impairment.

Embryonic fibroblasts from Q111-Htt knock-in HD mice showed an increased number of lipid droplets and markedly reduced the association of lipid droplets with HD autophagosomes [260], which may contribute for impaired intracellular lipid storage observed in HD.

PolyQ-HTT aggregates also sequestered mTOR, as observed in COS-7 cells expressing mutant (Q74) huntingtin exon 1, brain tissue of mice expressing mHTT (Q82), and brain tissue from HD patient’s caudate and putamen [271]. Additionally, ULK1 activity is also decreased in the brains of zQ175 HD mice, since ULK1 substrates such as Beclin-1 and ATG14 are less phosphorylated, together with the redistribution of ULK1 to an insoluble fraction where aggregated mHTT was found [269]. Moreover, Metzger and coworkers showed that the V471A polymorphism in ATG7 is related to an earlier onset form of HD [290].

As described before in this review, TFEB is a master regulatory transcription factor of the autophagy–lysosome pathway (ysosomal biogenesis and autophagy), which was shown to be upregulated by peroxisome proliferator-activated receptor-gamma coactivator (PGC)-1alpha [291], a member of transcription coactivators that plays a central role in the regulation of cellular energy metabolism, mitochondrial biogenesis, and adaptive thermogenesis. N171-82Q HD transgenic mice showed abnormal TFEB expression and activity, suggesting that TFEB signaling is impaired in HD [291]. Concordantly, TFEB can promote polyQ-HTT clearance [291], which emphasizes TFEB as a possible therapeutic target for HD or other diseases with protein aggregates accumulation.

Therefore, it is necessary to further understand autophagy dysfunction in HD, since preventing mHTT accumulation and/or improving neuronal proteostasis may constitute relevant therapeutic opportunities in HD.

#### 3.3.2. Mitophagy in HD

Previous studies reported ultrastructural defects in mitochondria isolated from post mortem HD cortical tissue and compromised oxidative function and ATP synthesis in pre-symptomatic HD carriers [292], suggesting that mitochondrial dysfunction is an early relevant pathogenic mechanism. HD mutant cells and striatal and cortical neurons isolated from YAC128 transgenic mice presented altered mitochondrial bioenergetics, including pyruvate dehydrogenase (PDH) dysfunction [293], as well as Δψm deregulation [294], augmented mitochondrial ROS production [295], and increased Ca^2+^ uptake in isolated mitochondria from pre-symptomatic R6/2 and YAC128 brain mice [296]. We further showed similar mitochondrial results in HD human lymphoblasts [297]. Profound changes in Δψm associated with apoptotic events and reduced ATP levels were also observed in symptomatic HD cybrids (an ex-vivo peripheral model obtained from the fusion of HD human platelets with mtDNA-depleted rho0 cells) and in HD human B-lymphocytes [298,299,300]. Concordantly, when compared with WT cells, striatal ST*Hdh*^Q111/Q111^ cells showed significant Δψm reduction after increasing Ca^2+^ concentrations [301]; in the case of primary striatal neurons derived from YAC128 mice, reduced Δψm and reduced mitochondrial Ca^2+^ handling occurred due to cytosolic Ca^2+^ overload after selective activation of *N*-methyl-d-aspartate (NMDA) receptors [302]. Furthermore, abnormal mitochondrial morphology and trafficking were observed in post mortem HD patients’ brain specimens and HD human lymphoblasts [303]. We previously showed that HD patients’ mitochondrial platelets presented reduced the activity of citrate synthase in pre-symptomatic and Cx-I in pre-symptomatic and symptomatic HD carriers [304]. In accordance with thi, isolated brain mitochondria from caudate nucleus of HD patients [305] and from different HD cells (human neuroblastoma cells; ST*Hdh*^Q111/Q111^ striatal cells), animal models (Hdh(CAG)150 knock-in mouse) showed HTT fragments in close contact with mitochondria [304,306], suggesting a direct effect of mHTT on mitochondrial function. Indeed, mHTT may cause direct effects by physically interacting with the organelle and associated proteins and further indirect effects on mitochondria and energy metabolism via transcriptional deregulation (e.g., by interfering with PGC-1alpha transcriptional activity). Thus, abnormal mitochondrial membrane potential (Δψm), oxidative stress, impaired oxidative phosphorylation (OXPHOS), and loss of mitochondrial dynamics, accompanied by mitochondrial morphology modifications and/or decreased biogenesis, favor the accumulation of damaged mitochondria in HD [307].

Abnormal elimination of dysfunctional mitochondria observed in HD suggests an impairment in mitophagy. Levels of basal mitophagy were seen to be reduced in the DG of HD mice crossed with the mito-Keima mouse line [308]. Moreover, a recent study showed that mitophagy is affected in ST*Hdh*^Q111/Q111^ striatal cells [309]. These authors showed that mHTT affects the initiation of the mitophagy process, as well as the recruitment of mitophay receptors and its interaction with LC3-II during mitophagy [309].

As explained earlier, PINK1-Parkin-mediated mitophagy starts with PINK1 stabilization at the OMM of damaged mitochondria to recruit Parkin. In the caudate nucleus from HD patients, mRNA expression of PINK1 is significantly decreased [310]. Augmented mitochondrial fragmentation [311] and inefficient incorporation of mitochondria into autophagosomes [265] are HD features; thus, decreased PINK1 may exacerbate the dysfunctional autophagosome recruitment in HD cells. Concordantly, PINK1 overexpression in HD flies and ST*Hdh*^Q111/Q111^ cells proved to be protective in these models [312]. Moreover, juvenile HD fibroblasts showed increased Parkin levels [313]. Although there is some lack of information regarding the alterations in PINK1/Parkin-dependent mitophagy in HD, its activation may constitute an interesting therapeutic approach. Moreover, mHTT can interact with Drp1, resulting in increased Drp1 GTPase activity and consequently increased mitochondrial fission, causing reduced mitochondrial function [303]. Recently, Aladdin and co-workers showed that skin fibroblasts from juvenile HD patients had significantly lower levels of mitochondrial fusion and fission proteins and reduced branching in the mitochondrial network. Furthermore, juvenile HD fibroblasts revealed higher proteasome activity, which was associated with elevated gene and protein expression of Parkin, as well as increased proteasomal degradation of the mitochondrial fusion protein Mfn1 in diseased cells [313]. These data suggest that expansion of mHTT is linked to increased proteasomal activity and faster turnover of specific substrates of the ubiquitin–proteasome system in order to protect cells, which could contribute to altered mitochondrial dynamics in early phases of the disease.

Additionally, besides mitophagy, mHTT disturbs mitochondrial trafficking, since mHTT aggregates may physically obstruct intracellular organellar dynamics, whereas diffuse mHTT can also interfere with trafficking adaptor proteins, affecting mitochondrial and autophagosomal dynamics [314]. An expected consequence of mHTT-induced mitochondrial dysfunction is a decrease in ATP production, restricting ATP-dependent activities of the proteostasis network [315]. Besides ATP limitation, mitochondrial dysfunction can damage the autophagic component of proteostasis by other mechanisms. For example, mitochondrial dysfunction might disrupt ER-mitochondrial contact sites, which compromises autophagosome formation [316]. Likewise, mitochondrial dysfunction is related to abnormal mitochondria–lysosome reciprocal regulation [316] and impaired lysosomal function [317], which may compromise the completion of autophagy.

## 4. Therapeutic Strategies

### 4.1. Targeting Macroautophagy and Mitophagy in AD

Current AD therapies largely delay the symptoms without targeting the pathogenic mechanisms. The presence of misfolded proteins and impairment of autophagy are two relevant causative factors in AD; thus, autophagy constitutes a possible therapeutic approach for AD. Although it is speculative that induction of autophagy might be a promising therapeutic strategy, the disruption of several steps of autophagic flux described in AD complicates this assumption. Moreover, caution must be taken when studying the effect of autophagy-targeting drugs in the different phases of disease progression.

A possible mechanism to activate autophagy is through the modulation of upstream regulatory signaling pathways towards autophagy induction. The mTORC1 pathway is a well-established signaling player in autophagy regulation and is dysregulated in AD, establishing a possible therapeutic target. Rapamycin is an FDA-approved drug that inhibits mTORC1 activity, resulting in autophagy activation. Long-term treatment with rapamycin rescued cognitive deficits in several mouse models of AD [318]. The beneficial effects mediated by rapamycin include decreasing Aβ deposition and reducing phosphorylation and accumulation of misfolded tau into NFTs [319,320]. SMER28, an enhancer of rapamycin, increased autophagy by an ATG5-dependent pathway and reduced Aβ peptide accumulation [321]. Notably, rapamycin-mediated improvements were observed when the treatment was initiated before symptoms onset [322]. Consistently, autophagy induction with rapamycin was not effective in ameliorating cognition if Aβ plaques and tangles were already present [322]. In late stages of the disease, the lysosomal system is severely compromised, and rapamycin treatment might even exacerbate the pathology. Despite all the preclinical evidence showing that modulation of mTORC1 with rapamycin is a promising therapeutic strategy at early stages of AD, no clinical trial was carried out so far [323]. It is noteworthy to highlight that mTOR is a central hub of intracellular signaling, including regulation of gene translation and cell homeostasis. It is reasonable to speculate that long-term inhibition of mTOR might have side toxic effects, so it is urgent to develop novel autophagy modulators. Another promising strategy is the genetic modulation of autophagy-related proteins that are disturbed in AD. For instance, a study showed that injection of a lentivirus encoding beclin-1 in hippocampus and frontal cortex of APP transgenic mice induced autophagy and reduced intracellular and extracellular Aβ deposition [113]. Moreover, increasing p62 expression in an APP/PS1 mouse model resulted in autophagy activation by a mTOR-independent pathway, leading to a reduction of Aβ pathology and amelioration of cognitive deficits [324]. Genetic-based strategies to modulate autophagy might constitute a more accurate therapy in the AD context, but a deeper characterization of all the autophagic steps and proteins affected in AD is required prior to therapeutic strategies’ implementation.

Carbamazepine (CBZ) is an anti-epileptic drug that showed promising results in the management of agitative behavior in AD patients [325]. The CBZ was found to inhibit mTORC1 activity, thus enhancing autophagy and decreasing amyloid burden and Aβ_42_ levels in transgenic AD mouse models [326]. Nevertheless, its secondary interactions with other drugs make it less popular among anticonvulsants used in the context of AD.

Memantine is an antagonist of the *N*-methyl-d-aspartate (NMDA) receptor and one of the few FDA-approved drugs for symptomatic treatment of moderate to severe AD. Some studies demonstrated its efficiency in ameliorating synaptic function, brain amyloidosis, and memory decline [327]. A study showed that memantine might exert its function by inhibition of autophagy through mTORC1 or Beclin-1 signaling in AD [328]. Contrariwise, memantine was identified as an autophagy enhancer on a large screening study to characterize clinically approved molecules for age-related disorders and neurodegeneration [329]. Combination therapy of memantine with cholinesterase inhibitors has been used in moderate to severe AD cases and showed improvement of cognition [330], but more studies are required to clarify the mechanism of action of memantine as an autophagy modulator in AD.

Metformin is an antidiabetic drug that activates AMPK by increasing AMP levels and has been used in diabetes type II patients to prevent dementia and Alzheimer-like phenotypes associated with hyperinsulinemia [331]. The use of metformin as monotherapy for AD is contradictory. AMPK activation suppresses mTOR activity and induces autophagy, thus exacerbating autophagosomes accumulation and also Aβ generation by γ- and β-secretase [332,333]. Metformin was found to aggravate Aβ pathology through increased Aβ generation by transcriptional up-regulation of BACE1 and Aβ secretion in neuronal cells models [332]. A very promising work showed that metformin is able to increase microglial phagocytosis and lysosomal acidification, enhancing extracellular Aβ clearance and preventing plaque formation [334], which may compensate Aβ generation and secretion in neuronal cells. Other studies showed that metformin decreases hyperphosphorylation of tau through an AMPK-independent mechanism [335]. Further research demonstrated that metformin increases the levels of insoluble tau species despite having a beneficial effect on the hyperphosphorylation state of tau [336]. The overall studies performed so far to characterize the effect of metformin in AD have generated conflicting results [337]. These discrepancies are probably an outcome of the effect of metformin on distinct biological pathways (reviewed in detail in [338]) and further research is required to address if metformin should be used as an AD therapeutic drug targeting autophagy.

The use of natural bioactive compounds extracted from food to modulate biological processes have been on the spotlight for the last years. One of the best characterized targets is AMPK, both in neurodegenerative and metabolic disorders. Resveratrol, a polyphenol found in red grapes and a recognized SIRT1 activator, was shown to stimulate AMPK in neurons [339]. Additionally, resveratrol lowers Aβ levels and secretion through AMPK-mediated inhibition of mTOR activity and an increase of lysosomal degradation in mouse primary neurons [340]. Moreover, dietary supplementation with resveratrol lowered amyloid plaque formation and rescued memory loss in transgenic AD mouse models [341]. Resveratrol can indirectly activate SIRT1 and promote clearance of abnormal proteins through mTOR-dependent and independent mechanisms [342]. In a 52-week phase II clinical trial, resveratrol was orally administered to AD patients with mild-to-moderate dementia and was detected in low nanomolar concentrations in CSF of these patients, meaning this compound can cross the BBB but with poor bioavailability. Surprisingly, Aβ_40_ levels were present in CSF and plasma of placebo-control groups at lower levels compared to the resveratrol-treated group at the final phase of the study [343], supporting the crossing through the BBB and a central effect on the brain. However, a positive effect of resveratrol on AD biomarkers such as CSF and plasma Aβ42 or p-tau was not detected in this pilot study, possibly due to the small size sample. Nevertheless, resveratrol might have beneficial effect on AD through the activation of autophagy by SIRT1 and mTOR-mediated signaling pathways. Quercetin is a flavonol mainly found in black and green tea that can also activate AMPK. Recent studies showed that quercetin administration reduced Aβ deposition and tau hyperphosphorylation and ameliorated cognitive function in a triple transgenic AD mouse model [344]. Moreover, long-term treatment with quercetin was shown to be preventive in neurodegeneration if administered prior to the appearance of AD histopathological alterations in the same model [344]. The possible modulation of autophagy by resveratrol or quercetin in AD supports further research as therapeutic strategies.

Modulation of GSK3 activity may also constitute a new therapeutic strategy for AD [345]. GSK3β regulates phosphorylation levels of tau and PSEN and can also modulate lysosomal acidification and reduce Aβ production [346]. Curiously, it was proposed that a beneficial effect of GSK3β inhibition occurs by reactivation of mTOR and repression of autophagy, which, in turn, ameliorate cognitive functions in 5xFAD mice model. Other studies contested the protective effect of mTOR modulation in AD; activation of mTOR improves cognitive deficits and was associated with a reduction in Aβ-associated events [347,348]. Lithium, a mood stabilizer used for bipolar disease, has been tested as a potential therapeutic drug for AD [349,350]. Lithium regulates several biochemical processes, among them the inhibition of GSK3β and modulation of the phosphatidylinositide cascade [351]. The intake of lithium by mutant tau transgenic mice delayed the formation of neurofibrillary tangles [352] and reduced Aβ pathology in the *Drosophila* AD model [353]. Moreover, lithium is a positive regulator of autophagy both by an mTOR-dependent pathway [354] and by depletion of intracellular free inositol and 1,4,5-inositol trisphosphate (IP_3_) [355], possibly through the inhibition of the IP_3_ receptor signaling cascade, a distinct autophagy-modulatory mechanism [356]. A clinical trial is currently ongoing to evaluate the effect of lithium on the onset of dementia in elderly people and correlation with AD progression (Clinical trial: NCT03185208).

Long-term treatment with nicotinamide reduced the accumulation of Aβ and hyperphosphorylated tau in the cortex and hippocampus of 3xTg-AD model mice with attenuation of cognitive decline [357]. This was accompanied by improvement of lysosomal acidification, autophagic–lysosomal function, and reduction of LC3-II, which indicates improvement of autolysosomal degradation. Consistently, another study with a 3xTg-AD mice model showed that nicotinamide administration was able to modulate SIRT1 activity and lysosomal proton gradient, thus promoting autolysosome acidification [358]. Despite its apparent beneficial effects, nicotinamide showed good tolerance but limited efficacy in cognitive improvement in a phase II clinical trials with AD patients after 24 weeks of treatment (Clinical trial: NCT00580931) [359]. Nevertheless, other clinical trials were approved to evaluate nicotinamide’s effect on tau levels in CSF of AD patients during a 48-week period (Clinical trials: NCT03061474), but no results have been published so far. Despite potential beneficial effects, nicotinamide that is released from nicotinamide adenine dinucleotide (NAD+, a nucleotide required for sirtuins activity) during the deacylation reaction of sirtuins was shown to act as a non-competitive inhibitor [360] and thus may abrogate neuroprotective effects exerted by these class III lysine deacetylases.

Because the late steps of autophagic flux are also compromised in AD, it is possible that therapeutic targeting of early steps may not be sufficient to fully restore the mechanism and alleviate protein aggregate accumulation. Therefore, an interesting target for drug development is TFEB, which modulates autophagosome and lysosome biogenesis and is also a regulator of autophagy [149]. It was shown that overexpression of TFEB reduced Aβ accumulation and the presence of phospho-tau in tangles and ameliorated cognitive and synaptic deficits in both the rTg4510 and APP/PS1 mouse model of AD [361]. Another study showed that injection of TFEB into the hippocampus of APP/PS1 transgenic mice by an adeno-associated virus promoted its translocation into the nucleus and enhanced lysosomal function and autophagy, which lowered Aβ levels and amyloid plaques [155]. Moreover, activation of astroglial TFEB resulted in the clearance of extracellular tau through lysosomal degradation, contributing to the reduction of tau spreading [362], which supports a neuroprotective effect of TFEB modulation in AD tau pathology. Therapeutics targeting TFEB activation should consider avoidance of interfering with mTOR activity and related signaling pathways. The search for new TFEB-targeting molecules has increased in the last years with the goal to attenuate Aβ generation and amyloid plaque deposition in AD.

Since neuronal mitophagy is compromised in AD, the discovery of new mitophagy inducers constitutes a promising therapeutic approach to promote clearance of damaged mitochondria and associated pathogenic hallmarks of the disease [363]. Modulation of autophagy, particularly the late steps, is expected to enhance mitophagy in AD. For instance, metformin stimulates mitophagy by restoring mTOR-dependent activation of autophagy and the Parkin-mediated mechanism [364]. Another study showed that modulation of autophagy with Nilotinib promotes the increase of intracellular levels of Parkin that interacts with Beclin1, reducing Aβ accumulation [365]. Nevertheless, is still unknown if the modulation of Parkin levels by Nilotinib also promotes mitophagy in AD.

Modulation of the NAD^+^/sirtuins pathway is a potential therapeutic target that is expected to improve mitophagy in AD. A recent drug-screening study showed that a combination of NAD^+^ precursors with mitophagy inducers, urolithin A and actinonin, restored mitophagy and ameliorated cognitive impairment by reducing phospho-tau and Aβ deposition in AD transgenic nematodes and mouse models [163]. Supplementation with NAD^+^ precursors attenuated Aβ and tau pathological hallmarks in AD models in part via the activation of mitophagy [163].

Overall, the conflicting data demonstrate that the induction of autophagy as a generalized therapy for AD is questionable. Stimulation of autophagy is not always beneficial and might even exacerbate Aβ pathology, resulting in elevated Aβ production and secretion. Autophagy is regulated by a complex crosstalk network of upstream signaling pathways that also regulate other cellular processes. The autophagy-inducing effect of each drug targeting one of these regulatory signaling pathways or proteins will most probably have an effect on the other signaling branch with compensatory or opposite effects. Additionally, some autophagy-targeting therapies directly modulate Aβ generation. The differences among AD models might also complicate the conclusions about autophagy modulation and/or Aβ- and tau-associated pathologies and consequent improvement of cognitive deficits. Additionally, the lack of a standard method to measure autophagy in vivo also compromises the clinical assessment of the therapeutic efficiency of drugs targeting autophagy in patients.

**Figure 4:**
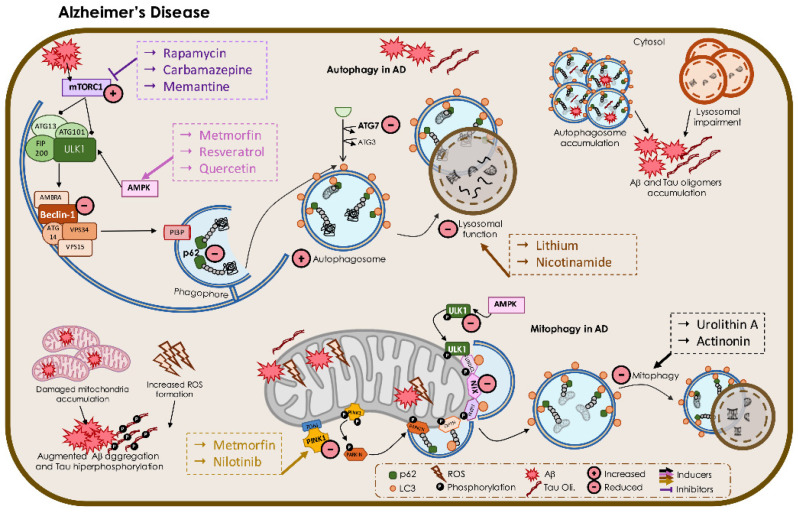
Overview of altered regulation of autophagy and mitophagy mechanisms in AD—role of protective compounds. Autophagy activity and related proteins are decreased in AD. mTORC1 was shown to be increased in AD, whereas its inhibitors (referred to in the purple dashed box) were shown to have positive effects in different AD models, most of them through diminished mTORC1 levels. Concordantly, AMPK inducer agents (referred to in the pink dashed box) were also shown to have positive effects in AD models. Several AD models showed increased autophagosome accumulation and reduced lysosomal function, which results in Aβ and Tau oligomer accumulation. Moreover, inducers of lysosomal function (referred to in the brown dashed box) were shown to significantly improve AD features in different AD models. Regarding augmented damaged mitochondria, AD models showed reduced mitophagy activity, whereas the inducer agents (referred to in the black dashed box) were also shown to increase mitophagy in AD models. Additionally, PINK1 levels were shown to be reduced in AD models, while the inducer agents (referred to in the yellow dashed box) were shown to increase those levels with positive effects.

In conclusion, therapeutic development targeting autophagy should take into account the multifactorial causes of AD. Thus, the rational design of AD therapeutic drugs or the identification of combined therapeutic strategies should consider targeting both autophagy and APP processing or Aβ generation and tau pathology. The exact autophagy defect, when to use autophagy-modulating therapies during the disease progression, and the duration of the treatment should all be considered when developing novel therapeutic strategies for AD (Figure 4).

### 4.2. Targeting Macroautophagy and Mitophagy in PD

Growing evidence provided by PD models reveals that the autophagic machinery is deeply hampered in PD, so macroautophagy emerges as a relevant therapeutic target to promote the clearance of toxic aggregates. For instance, administration of rapamycin is sufficient to protect dopaminergic neurons from degenerating in mice subjected to MPTP-induced parkinsonism [366]. The protective effect of rapamycin is associated with increased autophagy, since mice exposed to MPTP with prior rapamycin treatment had no observable accumulation of late autophagosomes, unlike the group that was exposed solely to MPTP. In another model of PD, using 6-OHDA-treated mice, rapamycin was also effective in restoring motor impairment with decreased aSyn deposition [367]. Likewise, Crews and co-workers elegantly demonstrated that intra-cerebral infusions of rapamycin in the cortical layer of mice overexpressing WT aSyn mitigated the anomalies observed in the autophagic process, such as LC3 and cathepsin D expression, along with augmented clearance of aSyn deposits [368].

A study using metformin to enhance AMPK1-dependent autophagy demonstrated that this drug was able to rescue motor impairment and the proinflammatory profile of MPTP-treated mice, through an increased activity of autophagy and the rescue of the autophagic flux [369]. Although these studies highlight the importance of autophagy to clear aSyn deposits and to halt the neurodegenerative process, upstream regulation of autophagy produces many undesirable effects, which affect treatment efficacy in many disease models [370]. Beclin-1, for instance, is essential in the initial steps of autophagosome formation and thus plays a key role in controlling autophagy. Effectively, Beclin-1 lentiviral injection in mice overexpressing aSyn rescues the negative impact of aSyn pathology in the cortical layer of the brain by ameliorating the number of LC3-positive vesicles and consequently enhancing the clearance of aSyn aggregates present in these mice [371]. In consonance with this study, the activation of Beclin-1-dependent autophagy with isorhynchophylline induced the degradation of aSyn oligomers in dopaminergic neurons constitutively overexpressing the A53T aSyn mutant form [372]. Mitophagy is likely to have a significant contribution, since Beclin-1 activity contributes to the translocation of Parkin to the OMM for the initiation of mitophagy [373].

As described before in this review, the removal of damaged mitochondria by mitophagy is highly significant for normal neuronal function. Indeed, an increasing body of evidence demonstrates a therapeutic value of removing damaged mitochondria in several PD models [374]. Pharmacological activation of PINK1 through the treatment of dopaminergic SH-SY5Y cells with a neo-substrate, kinetin triphosphate, increases the extent of phosphorylation of its substrates and promotes a faster recruitment of Parkin to mitochondria, resulting in an amelioration of the negative impacts caused by oxidative stress [375]. Additionally, an interesting study using *Drosophila melanogaster* demonstrated that, by overexpressing PINK1, the removal of damaged mitochondria improves through higher facilitation of mitochondrial axonal motility [376]. Consistent with this approach, overexpression of Parkin is beneficial in a MPTP mouse model. Transgenic mice overexpressing Parkin are much less susceptible to MPTP-induced dopaminergic neurodegeneration and present lower levels of aSyn oligomers in striatal homogenates [377]. Likewise, flies who were administered with SR3677 a ROCK inhibitor that promotes mitophagy enhancement through Parkin activation demonstrated alleviated motor behavior after a paraquat insult. ROCK is consistently activated in several neurodegenerative models and is also considered to be an autophagy modulator [378]. Remarkably, damaged mitochondria are driven for lysosomal degradation upon SR3677 treatment [379].

CMA is also responsible for maintaining the levels of native aSyn. A fascinating study demonstrated that animals overexpressing WT aSyn receiving a stereotaxic injection of lentiviral LAMP2a displayed higher CMA activity concomitant with decreased levels of endogenous aSyn than untreated mice [380]. MicroRNAs (miRNAs) can also modulate CMA events. Interestingly, miRNAs miR-26b, miR-106a, miR-301b and miR-21, miR-224, and miR-373, highly expressed in the SNpc of PD patients, are associated with decreased levels of Hsc70 and LAMP2a, respectively [381]. Furthermore, modulation of CMA by using geniposide has demonstrated interesting results in rescuing dopaminergic neurodegeneration in a MPTP-induced mouse model by increasing LAMP2a expression and reducing the levels of endogenous aSyn through the inhibition of miR-21 [382].

**Figure 5:**
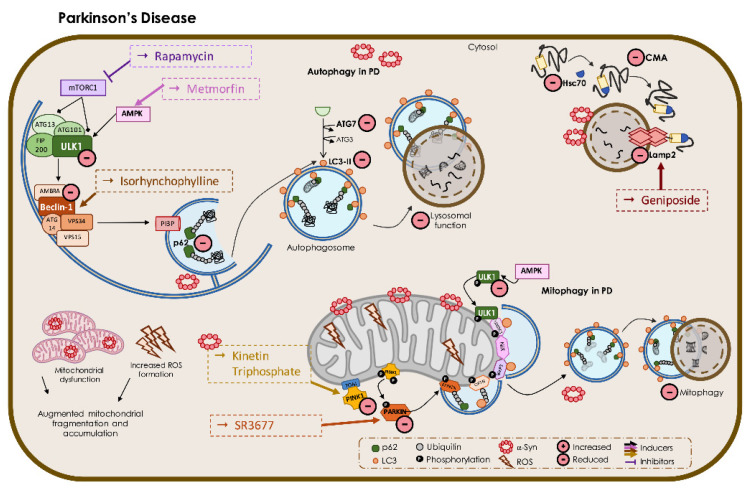
Overview of altered regulation of autophagy and mitophagy mechanisms in PD—role of protective compounds. Autophagy activity is altered in PD. The mTORC1 inhibitor and the AMPK inducer were shown to be protective in PD (referred to in purple and pink dashed boxes, respectively). Concordantly, the Beclin-1 inducer agent (referred to in the brown dashed box) was shown to have positive effects in PD models favoring autophagy. Several PD models showed reduced CMA function, whereas the LAMP2A inducer (red dashed box), was shown to significantly improve CMA function and PD features. Furthermore, augmented dysfunctional mitochondria and reduced mitophagy were observed in several PD models. Concordantly, PINK1 and PARKIN were shown to be diminished in PD, whereas inducer agents (referred to in the yellow and orange dashed boxes, respectively) were shown to have positive effects in different PD models, most of them by increasing mitophagy activity.

Regarding these observations, we can appreciate that autophagy naturally has an imperative function in PD since it can abrogate the main cellular defects occurring in diseased neurons when properly approached. Hence, pharmacological intervention or gene therapy that facilitates mitochondrial degradation seem to be at the forefront of disease-modifying therapies to treat PD or at least halt disease progression, which is critical since no effective treatment exists yet (Figure 5).

### 4.3. Targeting Macroautophagy and Mitophagy in HD

Considering the critical role of autophagy in mHTT clearance, the therapeutic study of this mechanism in HD is evident. Pharmacological or genetic therapeutic manipulation of autophagy in vivo has shown outstanding results in animal models of HD, with improvement of behavioral motor abnormalities and neuropathology. Some approved drugs, such as rapamycin, rilmenidine, and spermidine, are effective autophagy inducers and have been used in these experiments. Recently, reducing HTT expression through DNA editing, siRNAs, shRNAs, or antisense oligonucleotides genetic approaches has been tested along with pharmacological treatment to lower mHTT protein levels.

Ravikumar and colleagues have shown that the autophagy activation by rapamycin, a mTOR inhibitor, has neuroprotective effects and attenuated HTT toxicity in a fly model of HD [271]. Additionally, the mTOR inhibitor, CCI-779, a rapamycin analog, improved motor phenotypes in HD transgenic HD-N171-N82Q mice and reduced polyQ-HTT aggregate load by inducing autophagy activation [262]. However, more recent studies have shown that the restoration of mTORC1 activity improved motor deficits and brain pathology in N171-82Q HD mice [383]. Furthermore, mTORC1 activity was reduced in the striatum of HD patients and also in the striatum of this HD mouse model.

Rilmenidine, an mTOR-independent macroautophagy inducer, diminished the levels of harmful mHTT fragments and enhanced motor phenotypes in the same HD mouse model [384]. Additionally, HD R6/2 transgenic mice treated with trehalose, a disaccharide shown to induce macroautophagy, presented alleviated toxicity, improved motor function, and extended lifespan [385]. HD patient fibroblasts treated with trehalose showed reversed neurodegenerative phenotypes induced by UPS inhibition [386]. Lithium, which reduces IP3 levels, was shown to help clearing mHTT in drosophila HD models [354]. Berberine, which can induce autophagy via AMPK activation, further showed efficacy in an N171-82Q HD mouse model [387]. Clonidine, a modulator of cAMP or IP3 to induce autophagy, is another autophagy-related molecule able to ameliorate phenotypes in mammalian cell, fly, and zebrafish HD models [388]. Calpain can be a target for increasing autophagic flux of mHTT; indeed, knock-down of calpain showed reduced mHTT aggregate burden in the HD Drosophila model, and similar results were observed in transgenic N171-82Q mice, which overexpressed calpastatin (CAST), the endogenous inhibitor of calpain [389]. Pramipexole, a dopamine receptor agonist, was able to activate autophagy probably by modulating cAMP signaling pathways in the R6/1 HD mouse model, reducing soluble mHTT levels [390]. Additionally, ST*Hdh*^Q111/Q111^ cells [391] and 109Q/109Q mouse striatal cells [392] treated with metformin (an AMPK activating inducer of autophagy, as described before in this review) alleviated mHTT-associated cytotoxicity. Metformin is used to treat type II diabetes, and HD patients who were monitored in the Enroll-HD study (a world-wide observational and longitudinal study of HD patients) and were already taking metformin showed improved cognitive staging when compared to patients without metformin-regimen [393]. The use of the Akt inhibitor 10-[4′-(N-diethylamino)butyl]-2-chlorophenoxazine (10-NCP) also decreased mHTT aggregates in the HD mouse striatum and reduced striatal neuronal death [394]. Recently, Siddiqi and colleagues showed that HD-N171–82Q mice treated with felodipine, an anti-hypertensive drug, displayed significant improved features with a pharmacokinetics similar to its conventional application in humans [395].

The efficiency of some of these small molecules to target the same pathways in vivo has not been confirmed yet. Thus, the use of combined therapies for alleviating mHTT associated toxicity, which simultaneously upregulates autophagy through both the mTOR-independent and -dependent pathways, seem to be quite appealing. Indeed, HD models’ combination treatments, such as trehalose–rapamycin [396] or lithium–rapamycin [354], showed positive effects. Additionally, another strategy is regulating the activity of the autophagy initiation through the ULK1 complex. WT ULK1 overexpression decreased insoluble mHTT levels in cell lines, suggesting that ULK1 kinase activity is one limiting factor for the autophagic clearance of mHTT [269]. Thus, changes in mHTT load due to direct modulation of ULK1 activity are an exciting hypothesis that is now possible to test with the recent development of ULK1 activators [326] and inhibitors [397].

Lowering mHTT levels and toxicity has been the subject of several recent studies. Indeed, increasing mHTT selective autophagy could be an effective strategy for mHTT clearance, which can be reached by increasing mHTT cargo delivery, identifying compounds that retain mHTT to the autophagy degradation system. Recently, Li and colleagues performed a non-biased screening and identified four mHTT-LC3 linker compounds, able of tethering mHTT to the autophagosome for an allele-selective clearance of mHTT [398]. Furthermore, Hodges and colleagues found that levels of the adaptor protein OPTN were significantly decreased in the caudate nucleus of HD patients, suggesting that the selective turnover of aggregated proteins could be selectively reduced [399]. Thus, augmenting cargo recognition by adaptor proteins, possibly through posttranslational modifications, might be an interesting HD therapeutic approach.

As explained before, CMA could preferentially target soluble, N-terminal fragments of polyQ-HTT. Since truncated forms of mHTT, which are cytotoxic, are found in brains of HD patients and HD animal models, the selective degradation of these truncated forms through CMA might represent a therapeutic opportunity in HD. Indeed, the cytosolic chaperone Hsc70 can bind and directly deliver mHTT to the lysosome via CMA, leading to selective degradation of mHTT and reduced toxicity in HD mouse [400] and fly models [401]. Concordantly, Hsp70 knockout aggravated the motor symptoms in the R6/2 HD mice [402], whereas the phytocompound azadiradione increased the expression of Hsp70, which resulted in reduced polyQ aggregates and rescue of ommatidia morphology in Drosophila eyes [403]. Moreover, R6/2 HD mice using a CMA-targeting adaptor molecule against polyQ-HTT showed an ameliorated disease phenotype [400].

**Figure 6:**
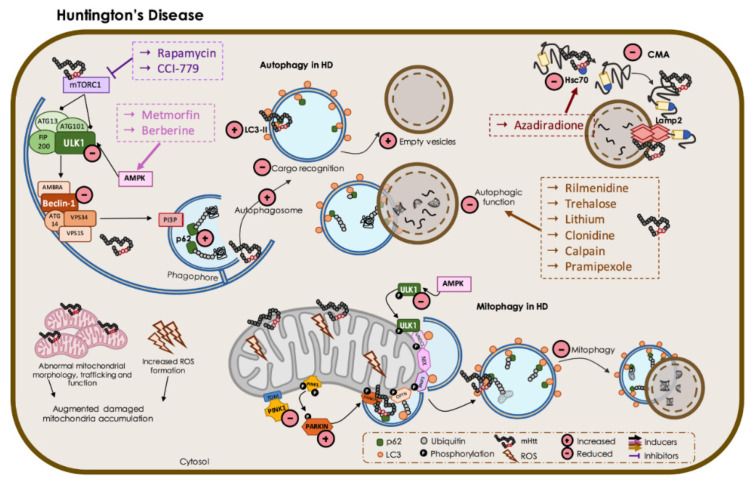
Overview of altered regulation of autophagy and mitophagy mechanisms in HD—role of protective compounds. Autophagic activity and related proteins are decreased in HD. Indeed, inducers of autophagic activity (referred to in the dashed box, in brown) were shown to significantly improve HD features in different HD models. The mTORC1 inhibitors and the AMPK inducers were shown to be protective in HD (referred to in the dashed boxes, in purple and pink, respectively), promoting autophagy. Several HD models showed increased autophagosome accumulation with reduced cargo recognition and accumulation of empty vesicles. Mitophagy is also impaired in several HD models.

Since there is cargo recognition failure when the expression of mHTT occurs, due to the physical interaction of HTT with p62 and ULK1 proteins, it has been proposed that mHTT could impair the delivery of flagged dysfunctional mitochondria to form autophagosomes. The PINK1/Parkin-dependent mitophagy pathway is the most well-characterized mitophagy pathway; however, PINK1/Parkin-independent mitophagy may also occur [404]. As described previously, normal HTT is an important protein in the control of autophagosome dynamics, along with huntingtin-associated protein 1 (HAP1), through the regulation of dynein and kinesin. Interestingly, impaired axonal transport and maturation of autophagosomes in the presence of mHTT was related to inefficient mitochondrial degradation [265]. These results suggest that inducing degradation of damaged mitochondria and favoring mitochondrial biogenesis are important therapeutic targets for HD treatment (Figure 6).

## 5. Conclusions

Accumulation of toxic intracellular protein aggregates (e.g., oligomers) is a hallmark of several neurodegenerative disorders, including AD, PD, and HD, and is believed to play a deleterious role in proteostasis and organelle function. Autophagy might constitute the main route for elimination of the aggregate-prone proteins or may be induced as a secondary clearance mechanism when other protein degradation processes fail throughout disease progression. Nevertheless, protein aggregate-lowering therapies have not always alleviated symptoms in different disease models, emphasizing that efforts for selective autophagy-based therapies focused on autophagic flux restoration, improvement of lysosomal function, and/or selectivity in aggregates delivery for autophagic clearance.

Indeed, in the last years, a significant increase in autophagy-targeted drug development has been observed, mainly due to improved knowledge of the mechanism and regulation of autophagy in different human diseases. Despite all the therapeutic strategies advances, caution must be taken when analyzing the effect of autophagy modulators in neurodegenerative disorders.

Although autophagy is a well-studied mechanism at biochemical and cellular levels, its exact role in neurodegeneration and disease progression is far from being understood. Indeed, there are some discrepancies reported in different disease models and/or different disease stages. In PD, for example, an initial increase in the autophagic process is observed in the early stage, while post-mortem samples point in the opposite direction. Although this may seem conflicting, the fact that several autophagic proteins often colocalize within the aSyn deposits suggests that the autophagic process starts to operate in an already established pathological state and decays throughout the progression of the disease.

Depending on the clinical context, induction or suppression of autophagy might constitute a possible therapeutic avenue. A similar approach can be applied to mitophagy, a major pathway of quality control mechanism for the removal of aged and defective mitochondria through lysosomal hydrolysis. Considering that mitochondrial dysfunction is largely involved in neuronal dysfunction, protection of mitochondrial function by improving mitophagy to maintain a healthy mitochondrial population within neurons might be an efficient strategy to promote neuroprotection and modify disease-related pathology (Table 1). Although mitophagy-related molecular and cellular mechanisms have been extensively studied in the last decade, abnormal mitophagy has only been recognized recently as a key player involved in neurodegeneration. Study of mitophagy status, as well as detailed mechanisms, are important for better understanding its role in disease pathogenesis. For example, activation of Parkin aimed to enhance mitophagy could be a promising strategy. Additionally, pharmacological agents capable of inducing mitophagy in order to augment clearance of damaged mitochondria might be an effective strategy for achieving a significant therapeutic benefit. Indeed, strategies to induce mitophagy by augmenting mild bioenergetic stress or inhibiting mTOR activity have also been proven to be advantageous in delaying or treating neurodegenerative disorders.

To date, there are very few studies showing clinical relevance when autophagy is modulated as a therapeutic strategy for these progressive and devastating diseases. Nevertheless, fundamental and preclinical studies suggest that modulators of autophagy constitute a promising therapeutic strategy as the mechanism is being unraveled in the context of neurodegenerative diseases. The lack of an autophagy-related biomarker that could be used to evaluate the clinical efficiency of the autophagy-modulating agent or a standard protocol to measure autophagy in vivo, among other unknown factors, has delayed the progress of therapeutic development.

The coming years are anticipated to foster the rationale for searching, screening, and characterizing novel autophagy-targeting drugs as therapeutic strategies in AD, PD, and HD, as increased knowledge is being gathered about the regulatory mechanisms and structural characterization of key targets that control autophagy. The design of selective modulators of autophagy or repurposing of drugs that may specifically target key autophagy intermediates will maximize therapeutic effect and minimize side effects. Novel therapeutic agents and genetic-based methodologies targeting autophagy will soon emerge as reliable therapies for neurodegenerative diseases.

## Figures and Tables

**Table 1:** Compounds-related pathway’s inhibitors or activators shown to exert beneficial effects in AD, PD, and HD models.

Disease	Protective Compounds-Related Pathways	Compounds	Models	References
Alzheimer’s Disease	mTORC1	Rapamycin	Tg2576 mice; Apolipoprotein E ϵ4 transgenic mice; an adeno-associated viral vector-based AD mice; AD hippocampal primary neurons; 3xTg-AD mice;	[318,319,320,321,322]
		Carbamazepine	AD patients;	[325]
		Memantine	AD patients; APP695swe expression in SH-SY5Y cells;	[327,328]
	AMPK	Metformin	AD patients; AD rat primary microglia; Tau transgenic mice primary neurons; human tau (P301S) transgenic mouse model;	[331,334,335,336]
		Resveratrol	AD primary neurons; Tg19959 mice; AD patients;	[340,341,343]
		Quercetin	3xTg-AD mice;	[344]
	Lysosomal Function	Lithium	AD patients; Mutant tau transgenic mice; Drosophila AD model	[349,350,352,353] Clinical trial: [NCT03185208]
		Nicotinamide	3xTg-AD model mice; AD patients;	[357,358,359] Clinical [trials: NCT03061474]
	PINK1	Nilotinib	C57BL/6 injected with Aβ_1–42_;	[365]
	Mitophagy	Urolithin A	APP/PS1 mice;	[163]
		Actinonin	APP/PS1 mice;	[163]
Parkinson’s Disease	mTORC1	Rapamycin	Dopaminergic neurons from MPTP-induced mice; 6-OHDA-treated mice; overexpressing WT aSyn mice;	[366,367,368]
	AMPK	Metformin	MPTP-treated mice;	[369]
	Beclin-1	Isorhynchophylline	Dopaminergic neurons with A53T aSyn overexpressing;	[372]
	PINK1	Kinetin triphosphate	Dopaminergic SH-SY5Y cells;	[375]
	Parkin	SR3677	Paraquat-treated fly model;	[378]
	Lamp2	Geniposide	MPTP-induced mice;	[382]
Huntington’s Disease	mTORC1	Rapamycin	HD fly model;	[271]
		CCI-779	HD-N171-N82Q mice;	[383]
	AMPK	Metformin	STHdh^Q111/Q111^ cells; 109Q/109Q mouse striatal cells; HD patients;	[391,392,393]
		Berberine	N171-82Q HD mouse model	[387]
	Hsc70	Azadiradione	HD fly model;	[403]
	Autophagic Function	Rilmenidine	N171-82Q HD mice	[384]
		Trehalose	HD R6/2 transgenic mice; HD patient fibroblasts;	[385,386]
		Lithium	Drosophila HD models;	[354]
		Clonidine	HD fly and zebrafish models;	[388]
		Calpain	HD Drosophila model; N171-82Q mice;	[389]
		Pramipexole	R6/1 HD mouse model;	[390]

## Data Availability

Not applicable.
